# Interaction between Cannabinoid Type 1 and Type 2 Receptors in the Modulation of Subventricular Zone and Dentate Gyrus Neurogenesis

**DOI:** 10.3389/fphar.2017.00516

**Published:** 2017-08-10

**Authors:** Rui S. Rodrigues, Filipa F. Ribeiro, Filipa Ferreira, Sandra H. Vaz, Ana M. Sebastião, Sara Xapelli

**Affiliations:** ^1^Instituto de Farmacologia e Neurociências, Faculdade de Medicina, Universidade de Lisboa Lisboa, Portugal; ^2^Instituto de Medicina Molecular, Faculdade de Medicina, Universidade de Lisboa Lisboa, Portugal

**Keywords:** postnatal neurogenesis, subventricular zone, dentate gyrus, cannabinoid type 1 receptor, cannabinoid type 2 receptor

## Abstract

Neurogenesis in the adult mammalian brain occurs mainly in two neurogenic niches, the subventricular zone (SVZ) and the subgranular zone (SGZ) of the dentate gyrus (DG). Cannabinoid type 1 and 2 receptors (CB_1_R and CB_2_R) have been shown to differently modulate neurogenesis. However, low attention has been given to the interaction between CB_1_R and CB_2_R in modulating postnatal neurogenesis (proliferation, neuronal differentiation and maturation). We focused on a putative crosstalk between CB_1_R and CB_2_R to modulate neurogenesis and cultured SVZ and DG stem/progenitor cells from early postnatal (P1-3) Sprague-Dawley rats. Data showed that the non-selective cannabinoid receptor agonist WIN55,212-2 promotes DG cell proliferation (measured by BrdU staining), an effect blocked by either CB_1_R or CB_2_R selective antagonists. Experiments with selective agonists showed that facilitation of DG cell proliferation requires co-activation of both CB_1_R and CB_2_R. Cell proliferation in the SVZ was not affected by the non-selective receptor agonist, but it was enhanced by CB_1_R selective activation. However, either CB_1_R or CB_2_R selective antagonists abolished the effect of the CB_1_R agonist in SVZ cell proliferation. Neuronal differentiation (measured by immunocytochemistry against neuronal markers of different stages and calcium imaging) was facilitated by WIN55,212-2 at both SVZ and DG. This effect was mimicked by either CB_1_R or CB_2_R selective agonists and blocked by either CB_1_R or CB_2_R selective antagonists, cross-antagonism being evident. In summary, our findings indicate a tight interaction between CB_1_R and CB_2_R to modulate neurogenesis in the two major neurogenic niches, thus contributing to further unraveling the mechanisms behind the action of endocannabinoids in the brain.

## Introduction

In the adult brain, new functional neurons are generated from neural stem/progenitor cells (NSPC), which have the ability to self-renew their own pool through cell proliferation and/or to generate cells of the neural lineage (neurons, astrocytes and oligodendrocytes) (Gage, 2000; Gross, 2000). This process, so called neurogenesis, occurs mainly in two restricted areas, the SVZ lining the lateral ventricles, and the SGZ within the DG of the hippocampus. In fact, SVZ and SGZ are regions rich in NSPC that originate neuroblasts, which then migrate toward their final destinations where they differentiate into mature neurons, a few being integrated into the neuronal circuitry (Lledo et al., 2006; Zhao et al., 2008; Ming and Song, 2011).

These neurogenic niches are highly regulated by several factors that dictate the NSPC rates of proliferation, differentiation, survival and maturation (Ming and Song, 2011). These factors play a crucial regulatory role under neuropathological conditions, in an attempt to balance the system, favoring the correct incorporation of newly formed neurons into the circuitry (Yoneyama et al., 2011). Therefore, understanding the molecular mechanisms and key elements that control the maintenance of neurogenic niches will contribute to the development of future potential therapies for brain disorders.

In recent years, an increasing interest has emerged on the role of endocannabinoids in neurogenesis. Cannabinoids act mainly on two types of receptors, type 1 and type 2 cannabinoid receptors (CB_1_R and CB_2_R). CB_1_R is considered the neuronal receptor whereas CB_2_R is considered the receptor of the immune system (Galve-Roperh et al., 2007), although increasing evidence shows that CB_2_R is present in several neuronal cell types (Onaivi, 2006; Savonenko et al., 2015). CB_1_R and CB_2_R are G-protein coupled receptors (GPCRs) predominantly targeted by anandamide (AEA) and 2-arachidonylglycerol (2-AG), the two major endocannabinoids in the CNS. These molecules target cannabinoid receptors differently: 2-AG acts as a full agonist of CB_1_R and CB_2_R whereas AEA acts a partial agonist for CB_1_R and CB_2_R (Luchicchi and Pistis, 2012). AEA and 2-AG are generated by the cleavage of membrane lipid precursors upon neural activity (on-demand synthesis model) (Ohno-Shosaku and Kano, 2014), and act as fast retrograde messengers to activate CB_1_R in presynaptic terminals and inhibit neurotransmitter release (Castillo et al., 2012; Mechoulam and Parker, 2013). Doing so, cannabinoid receptors regulate neurocircuitry dynamics, thus having important roles in many pathological processes such as anxiety, depression, feeding behavior, Parkinson’s disease, Alzheimer’s disease, neuroinflammation and pain (Kirkham, 2009; Katona and Freund, 2012; Di Iorio et al., 2013).

Besides their neuromodulatory role, endocannabinoids constitute a group of signaling cues that can regulate neurogenesis at several levels, including NSPC proliferation, differentiation, migration and survival, these actions being associated to either CB_1_R or CB_2_R (Aguado et al., 2005; Prenderville et al., 2015). In fact, CB_1_Rs and CB_2_Rs were shown to be expressed in rat SVZ and DG tissue and neurosphere-derived cells (Morozov and Freund, 2003; Gong et al., 2006; Arévalo-Martín et al., 2007). Importantly, CB_1_R activation was shown to promote proliferation in embryonic cultures, in SVZ neurosphere cultures and in the DG of 6-week old mice (Aguado et al., 2007; Hill et al., 2010; Andres-Mach et al., 2015). In the same way, activation of CB_1_R was also found to induce differentiation and maturation of *in vitro* NSPC (Compagnucci et al., 2013; Xapelli et al., 2013). Moreover, gathering evidence shows the implication of CB_2_R in processes related to the control of proliferation, differentiation, migration and survival of NSPC. Activation of CB_2_R was, indeed, shown to promote proliferation in embryonic cell lines, in SVZ neurosphere cultures and in the SVZ of young mice, as well as differentiation of human NSPC (Palazuelos et al., 2006, 2012; Goncalves et al., 2008; Avraham et al., 2014; Downer, 2014). Interestingly, it was recently shown that CB_2_R is necessary for neuroblast migration after stroke (Bravo-Ferrer et al., 2017).

Cannabinoid-based therapy may constitute a novel therapeutic strategy in the emerging field of brain repair. Indeed, some symptoms associated with adult brain disorders appear to be correlated with dysregulation of endocannabinoid signaling (Galve-Roperh et al., 2007; Mechoulam and Parker, 2013). Of particular interest, CB_2_R play an important role in neuro-immunomodulatory responses and, unlike CB_1_R, do not produce any psychoactive effects, thus being particularly promising targets to treat neuroinflammation-related brain disorders (Fernández-Ruiz et al., 2008; Rom and Persidsky, 2013).

Increasing evidence point to an important role of interactions between GCPR in the CNS, as well as to heteroreceptor formation (Ferré et al., 2007, 2014; González-Maeso, 2011; Ferré, 2015). In fact, CB_1_R were shown to modulate the release of several neuromodulators including dopamine, opioids, norepinephrine and others by interacting with other GPCRs, either by intracellular crosstalk of signal transduction or by forming heterodimers (Mackie, 2005; Demuth and Molleman, 2006; Navarro et al., 2008; Ferré et al., 2009b; Castillo et al., 2012). Similarly, crosstalk between CB_2_R and other GPCRs is also known to occur although the molecular and cellular basis for these interactions, the extent to which they occur and the impact on CNS function is still not fully understood (Callén et al., 2012; Balenga et al., 2014; Malfitano et al., 2014). Importantly, molecular and functional heteromerization of CB_1_R and CB_2_R has been shown for the first time in the study of Callén et al. (2012), where they demonstrated the presence of CB_1_R-CB_2_R heteromers in a variety of brain regions, depicting the existence of a bidirectional cross-antagonism phenomenon. Functional consequences of this heteromerization are poorly known.

Given the evidence that both CB_1_R and CB_2_R can affect neurogenesis as well as the evidence that CB_1_R and CB_2_R receptors may interact, we hypothesized that both receptors could act together to fine-tune neurogenesis. By testing this hypothesis, we now show for the first time that the action of cannabinoids on proliferation and differentiation of SVZ and DG NSPC involves a close interaction between CB_1_R and CB_2_R, suggesting a fundamental role of this crosstalk in the modulation of postnatal neurogenesis.

## Materials and Methods

### Ethics Statement

This study was carried out in accordance with the recommendations of “Directive 2010/63/EU.” The protocol was approved by the “iMM’s institutional Animal Welfare Body – ORBEA-iMM and the National competent authority – DGAV (Direcção Geral de Alimentação e Veterinária).”

### SVZ and DG Cell Cultures

SVZ and DG neurospheres were prepared from early postnatal (P1-3) Sprague-Dawley rats. SVZ and DG fragments were dissected out from 450 μm-thick coronal brain slices, digested with 0.05% Trypsin-EDTA (Life Technologies, Carlsbad, CA, United States) in Hank’s balanced saline solution (HBSS, Life Technologies), and mechanically dissociated with a P1000 pipette. The originated cell suspension was then diluted in serum-free medium (SFM), composed of Dulbecco’s modified Eagle’s medium/Ham’s F-12 medium with GlutaMAX (DMEM + GlutaMAX, Life Technologies) supplemented with 100 U/mL penicillin and 100 μg/mL streptomycin (Pen/Strep; Life Technologies), 1% B27 (Life Technologies) and growth factors (for SVZ cells: 20 ng/mL EGF; Life Technologies); for DG cells: 20 ng/mL EGF (Life Technologies) and 10 ng/mL FGF-2 (Life Technologies) (proliferative conditions). Single cells were then plated on uncoated Petri dishes at a density of 5,000 cells per cm^2^. SVZ cells were allowed to develop for six days, whereas DG cells were allowed to develop for twelve days, both in a 95% air-5% CO_2_ humified atmosphere at 37°C. Rat SVZ and DG neurospheres require different culture conditions, time length and growth factors, because DG-derived neurospheres need more time to reach optimal dimensions when comparing with SVZ (Guo et al., 2012) and SVZ cells do not require the growth factor FGF-2 to expand into neurospheres (Palmer et al., 1995; Kuhn et al., 1997; Woodbury and Ikezu, 2014). Six-day-old SVZ neurospheres and 12-day-old DG neurospheres were adhered for 24 h onto glass coverslips (at a density of approximately 60 neurospheres per well) coated with 0.1 mg/mL poly-D-lysine (PDL, Sigma–Aldrich, St. Louis, MO, United States) in SFM devoid of growth factors (differentiative conditions). One day after plating, the medium was renewed with or without (control) a range of pharmacological treatments for CB_1_R and CB_2_R ligands, chosen based on their affinity for CB_1_R and CB_2_R represented by *K_i_* value (**Table 1**).

**Table 1:** Pharmacological treatments used.

Drug	Biological activity	Concentration used	*K_i_* value, nM (according to Pertwee, 2008)	Catalog number	Company
**WIN55,212-2** [(R)-(+)-[2,3-Dihydro-5-methyl-3-(4-morpholinylmethyl)pyrrolo[1,2,3-de]-1,4-benzoxazin-6-yl]-1-naphthalenylmethanone]	Cannabinoid receptor CB_1_ or CB_2_ non-selective agonist	100 nM 300 nM 1 μM	1.89 to 123 for CB_1_R or 0.28 to 16.2 for CB_2_R	1038	Tocris, Bristol, United Kingdom
**ACEA** [*N*-(2-Chloroethyl)-5*Z*,8*Z*,11*Z*,14*Z*-eicosatetraenamide]	Cannabinoid CB_1_ receptor selective agonist	100 nM 300 nM 1 μM	1.4 for CB_1_R	1319	
**HU-308** [4-[4-(1,1-Dimethylheptyl)-2,6-dimethoxyphenyl]-6,6-dimethylbicyclo[3.1.1]hept-2-ene-2-methanol]	Cannabinoid CB_2_ receptor selective agonist	100 nM 300 nM 1 μM	22.7 for CB_2_R	3088	
**AM251** [*N*-(Piperidin-1-yl)-5-(4-iodophenyl)-1-(2,4-dichlorophenyl)-4-methyl-1*H-*pyrazole-3-carboxamide]	Cannabinoid CB_1_ receptor selective antagonist	1 μM	7.49 for CB_1_R	1117	
**AM630** [6-Iodo-2-methyl-1-[2-(4-morpholinyl)ethyl]-1*H*-indol-3-yl](4-methoxyphenyl)methanone]	Cannabinoid CB_2_ receptor selective antagonist	1 μM	31.2 for CB_2_R	1120	

### Pharmacological Treatments

To study cell proliferation, plated neurospheres were allowed to develop for 24 h (DIV 1) in the absence (control) or presence of CB_1_R and/or CB_2_R ligands (**Table 1**). Neuronal differentiation was assessed by allowing neurospheres to develop for 7 days (DIV 7) in the absence (control) or presence of the aforementioned ligands and an immunocytochemistry for different neuronal markers was performed. To study neuritogenesis, SVZ and DG neurospheres were dissociated using phosphate-buffered saline (PBS) without Mg^2+^/Ca^2+^ and with EDTA (2.5 mM KCl, 1.5 mM KH_2_PO_4_, 135 mM NaCl, 8 mM Na_2_HPO_4_, 0.5 mM EDTA.4Na) and plated in differentiative conditions at a density of 10000 cell/cm^2^. DG and SVZ cells were allowed to grow for 2 (DIV 2) and 3 days (DIV 3), respectively, in the absence (control) or presence of the aforementioned ligands and were then fixed and stained for βIII tubulin (neuron-specific class III beta-tubulin).

Whenever cultures needed to be co-treated with both agonists, SVZ or DG cells were primarily treated with CB_1_R (ACEA) selective agonist for 30 min prior to CB_2_R selective agonist (HU-308) treatment and then grown for 24 h or 7 days in the presence of the ligands (**Supplementary Figure S1A**). Similarly, treatment with the selective antagonist for CB_1_R (AM251) or CB_2_R (AM630) was performed for 30 min prior to the treatment with the non-selective cannabinoid receptor agonist WIN55,212-2 or CB_1_R (ACEA) or CB_2_R (HU-308) selective agonists and then co-incubated for further 24 h or 7 days (**Supplementary Figure S1B**). Additionally, whenever cultures needed to be treated with a combination of both antagonists and both agonists, SVZ and DG cells were firstly treated with selective antagonists for CB_1_R (AM251) and CB_2_R (AM630) for 30 min prior to CB_1_R selective agonist (ACEA), incubated for further 30 min before CB_2_R selective agonist (HU-308) treatment. Thereafter cells were allowed to grow for 24 h in the case of cell proliferation studies or 7 days in the case of neuronal differentiation studies (**Supplementary Figure S1C**).

### Cell Proliferation Study

To investigate the effect of the different pharmacological treatments on cell proliferation, SVZ and DG cells were exposed to 10 μM 5-bromo-2′-deoxyuridine (BrdU) (Sigma–Aldrich) for the last 4 h of each specific pharmacological treatment (24 h, DIV1). BrdU is a synthetic thymidine analog able to substitute thymidine in the DNA double chain synthesis occurring in dividing cells. Furthermore, neuronal differentiation of proliferating progenitors was assessed in SVZ and DG cells that were incubated in the absence (control) or in the presence of CB_1_R/CB_2_R ligands together with 10 μM BrdU for 24 h. Then the cells were washed and incubated without BrdU for more 6 days, fixed and co-stained for BrdU and a marker of mature neurons (neuronal nuclear protein, NeuN). For that, SVZ and DG cells were fixed in 4% paraformaldehyde (PFA), BrdU was unmasked by permeabilizing cells in PBS 1% Triton X-100 (Sigma–Aldrich) and DNA was denaturated in 1M HCl. Following blocking in PBS with 0.5% Triton X-100 and 3% bovine serum albumin (BSA), cells were incubated overnight with the anti-BrdU antibody and anti-NeuN antibodies (**Table 2**). Cells were incubated with the secondary anti-rat Alexa Fluor 488 and anti-rabbit Alexa Fluor 568 (**Table 2**). Nuclei were stained with Hoechst 33342 (6 μg/mL in PBS, Life Technologies). The final preparations were mounted using Mowiol fluorescent medium.

**Table 2:** Antibodies used for immunocytochemistry.

Antigen	Company	Catalog number	Host	Dilution
**Primary antibodies**				
βIII tubulin	Cell Signaling Technology	4466	Mouse	1:500
BrdU (5-bromo-2’-deoxyuridine)	AbD Serotec, Bio-Rad Laboratories (Oxford, United Kingdom)	OBT00306	Rat	1:200
CB_1_R (Cannabinoid receptor type 1)	Frontier Institute Co., Ltd (Japan)	CB1-GP-Af530	Guinea Pig	1:200
CB_2_R (Cannabinoid receptor type 2)	Santa Cruz Biotechnology	sc-293188	Mouse	1:200
Doublecortin (DCX)	Santa Cruz Biotechnology	sc-8066	Goat	1:200
Neuronal Nuclei (NeuN) (mature neuronal marker)	Cell Signaling Technology (Danvers, MA, United States)	12943	Rabbit	1:100
Nestin	Abcam (Cambridge, United Kingdom) Invitrogen	ab6142	Mouse	1:200
Vesicular GABA Transporter (VGAT)	Santa Cruz Biotechnology	sc-49574	Goat	1:200
Vesicular Glutamate transporter 1 (VGluT1)	Synaptic Systems	135302	Rabbit	1:100
Tyrosine Hydroxylase (TH)	Immunostar	22941	Mouse	1:100
Sox2	Merk Millipore (Massachusetts, EUA)	AB5603	Rabbit	1:200
**Secondary antibodies**				
Anti-Goat Alexa Fluor^®^ 488	Thermo Fisher Scientific (Rockford, IL, United States)	A-11055	Donkey	1:200
Anti-Rat Alexa Fluor^®^ 488	Thermo Fisher Scientific (Rockford, IL, United States)	A-21208	Donkey	1:200
Anti-Rabbit Alexa Fluor^®^ 568	Thermo Fisher Scientific (Rockford, IL, United States)	A-10042	Donkey	1:200
Anti-Goat Alexa Fluor^®^ 568	Thermo Fisher Scientific (Rockford, IL, United States)	A-11057	Donkey	1:200
Anti-Mouse Alexa Fluor^®^ 647	Thermo Fisher Scientific (Rockford, IL, United States)	A-21235	Goat	1:200

### Cell Differentiation Study

SVZ and DG cells treated for 7 days (DIV 7) were fixed in 4% PFA, permeabilized and blocked with 0.5% Triton X-100 and 6% BSA in PBS. Cells were then incubated overnight at 4°C with the primary antibodies against different neuronal markers in 0.1% Triton X-100 and 0.3% BSA (w/v) in PBS, and then with the appropriate secondary antibodies in PBS (**Table 2**). Nuclei counterstaining and mounting were performed as described previously.

### Western Immunobloting Analysis and Co-immunoprecipitation

Western blotting analysis of CB_1_R and CB_2_R was performed using SVZ and DG neurospheres that were plated and allowed to develop for 24 h (representing DIV 1) or 7 days (representing DIV 7) in SFM devoid of growth factors. The cells were harvested in RIPA lysis buffer [50 mM Tris-HCl (pH 7.5), 150 mM NaCl, 5 mM ethylenediamine, tetra-acetic acid (EDTA), 0.1%SDS and 1%Triton X-100, containing a protease inhibitor cocktail (pH 7.4, 4°C, Roche, Penzberg, Germany)]. Protein concentration was measured by the Lowry method and samples were treated with SDS-PAGE sample buffer [5x concentrated: 350 mM Tris, 10% (w/v) SDS, 30% (v/v) glycerol, 0.6M DTT, 0.012% (w/v) bromophenol blue], boiled 5 min at 95°C and processed for Western blotting analysis. All samples were applied with same amount of total protein and protein running was performed in SDS-PAGE gels (10% acrylamide/bisacrylamide for resolving and 5% for stacking gels), transferred to polyvinylidene difluoride (PVDF) membranes (GE Healthcare, Buckinghamshire, United Kingdom). Incubations with the primary antibodies against CB_1_R (1:500, Frontier Institute Co., Ltd, Japan) and CB_2_R (1:1000, Santa Cruz Biotechnology) were performed overnight at 4°C. Membranes were incubated with the respective secondary antibodies: goat IgG anti-guinea pig and goat IgG anti-mouse conjugated with horseradish peroxidase (HRP) (1:10000; Santa Cruz Biotechnology). For endogenous control of immunolabeling, PVDF membranes were reprobed with the antibody against actin (1:1000; #A2006, Sigma–Aldrich). Finally, immunoreactivity was visualized using ECL chemiluminescence detection system (Amersham-ECL Western Blotting Detection Reagents from GE Healthcare, Buckinghamshire, United Kingdom).

CB_2_Rs were immunoprecipitated from culture lysates using an antibody-bead mixture previously prepared. Rec-Protein *G*-Sepharose 4B Conjugate beads (Thermo Fisher Scientific) were first incubated with PBS 0.1% BSA for 1h at 4°C. After washing with PBS and centrifugation at 5000 rpm for 3 min, the pellet composed of beads was incubated for 4 h, at 4°C, with 1 μg of CB_2_R antibody. Following several washes, 35 μL of antibody-bead mixture was incubated with 250 μg of total protein (approximately 400 μL of culture lysate) overnight at 4°C. The mixture was centrifuged and the supernatant was collected. The remaining pellet of beads was washed three times with PBS 0.01% Tween 20 and re-suspended and denatured in 35 μL of SDS-PAGE sample buffer. Samples were boiled for 5 min at 95°C. Detection of CB_2_R and CB_1_R were performed through western blotting, as described above.

### Single Cell Calcium Imaging (SCCI)

To determine the functional neuronal differentiation of SVZ cells, the variations of intracellular calcium concentration ([Ca^2+^]_i_) in single cells following stimulation with 50 mM KCl and 100 mM histamine (Sigma–Aldrich) were analyzed as previously described for SVZ cells (Agasse et al., 2008). KCl depolarization causes an increase in [Ca^2+^]_i_ in neurons, whereas stimulation with histamine leads to an increase in [Ca^2+^]_i_ in stem/progenitor cells (Agasse et al., 2008; Xapelli et al., 2013). For SCCI analysis SVZ cultures plated in PDL-coated glass bottom cell culture dishes (Nest, NJ, United States) and grown for 7 days were loaded for 45 min with 5 μM Fura-2/AM (Invitrogen) and 0.02% pluronic acid F-127 (Invitrogen) in Krebs solution (132 mM NaCl, 4 mM KCl, 1.4 mM MgCl_2_, 1 mM CaCl_2_, 6 mM glucose, 10 mM HEPES, pH 7.4), in an incubator with 5% CO_2_ and 95% atmospheric air at 37°C. After a 5 min postloading period at RT in Krebs solution without Fura-2/AM and pluronic acid, to obtain a complete hydrolysis of the probe, the dish was mounted on an inverted microscope with epifluorescent optics (Axiovert 135TV, Zeiss) equipped with a xenon lamp and band-pass filters of 340 and 380 nm wavelengths. Cells were continuously perfused with Krebs solution and stimulated at defined periods of time by applying high-potassium Krebs solution (containing 50 mM KCl, isosmotic substitution with NaCl) and 100 μM histamine (**Supplementary Figure S2**). Throughout the experiments, the cells were continuously superfused at 1.5 mL/min with physiological solution. KCl and histamine were applied focally through a drug filled micropipette placed under visual guidance. Image pairs obtained every 200 ms by exciting the preparations at 340 and 380 nm were taken to obtain ratio images. Excitation wavelengths were changed through a high-speed wavelength switcher, Lambda DG-4 (Sutter Instrument, Novato, CA, United States), and the emission wavelength was set to 510 nm. Image data was recorded with a cooled CCD camera (Photometrics CoolSNAP fx) and processed and analyzed using the software MetaFluor (Universal Imaging, West Chester, PA, United States). Regions of interest were defined manually over the cell profile. KCl and histamine peaks given by the normalized ratios of fluorescence at 340/380 nm, at the proper time periods, were used to calculate the ratios of the responses.

### Microscopy

Fluorescence images were recorded using an LSM 880 confocal microscope or an Axiovert 200 inverted widefield fluorescence microscope (both from Carl Zeiss Inc., Göttingen, Germany), with a 40x objective. Images were recorded using the softwares ZEN 2.1 (black edition) or AxioVision 4 (both from Carl Zeiss Inc.).

### Statistical Analysis

In all experiments, measurements were performed at the border of SVZ and DG neurospheres, where migrating cells form a pseudo-monolayer of cells. In every independent experiment, which corresponds to one independent neurosphere culture from one litter, each condition was measured in three different coverslips. Percentages of BrdU, BrdU/NeuN, NeuN, DCX and DCX/NeuN immunoreactive cells were calculated from cell counts of five independent microscopic fields in each triplicated coverslip (representing *n* = 1) with a 40x objective (∼200–400 cells per field). For quantification of neuritogenesis neurites were manually traced using NeuronJ v1.4.3, a plugin from ImageJ v1.46r (10 random images were captured per glass coverslip, in triplicates), measuring the total neurite length, the maximal neurite length, as well as the number of primary dendrites and branch points. An average of at least 10 neurons were acquired per glass coverslip for each experiment. All experiments were analyzed in a double-blind fashion and part of data was normalized to each corresponding control.

For SCCI experiments, the percentages of neuronal-like responding cells (with a Hist/KCl ratio below 0.8) and of immature-like responding cells (with a Hist/KCl ratio between 1 and 1.3) were calculated on the basis of one microscopic field per glass coverslip, containing approximately 100 cells, in a total of 3–4 independent cultures where each condition are duplicate.

Data are expressed as mean ± standard error of the mean (SEM). Statistical significance was determined using one-way analysis of variance followed by Sidak’s or Dunnett’s-multiple comparison test, with *p* < 0.05 considered to represent statistical significance.

## Results

### Neurosphere Culture Characterization: SVZ and DG Cultures Exhibit Type 1 and Type 2 Cannabinoid Receptors

**FIGURE 1:**
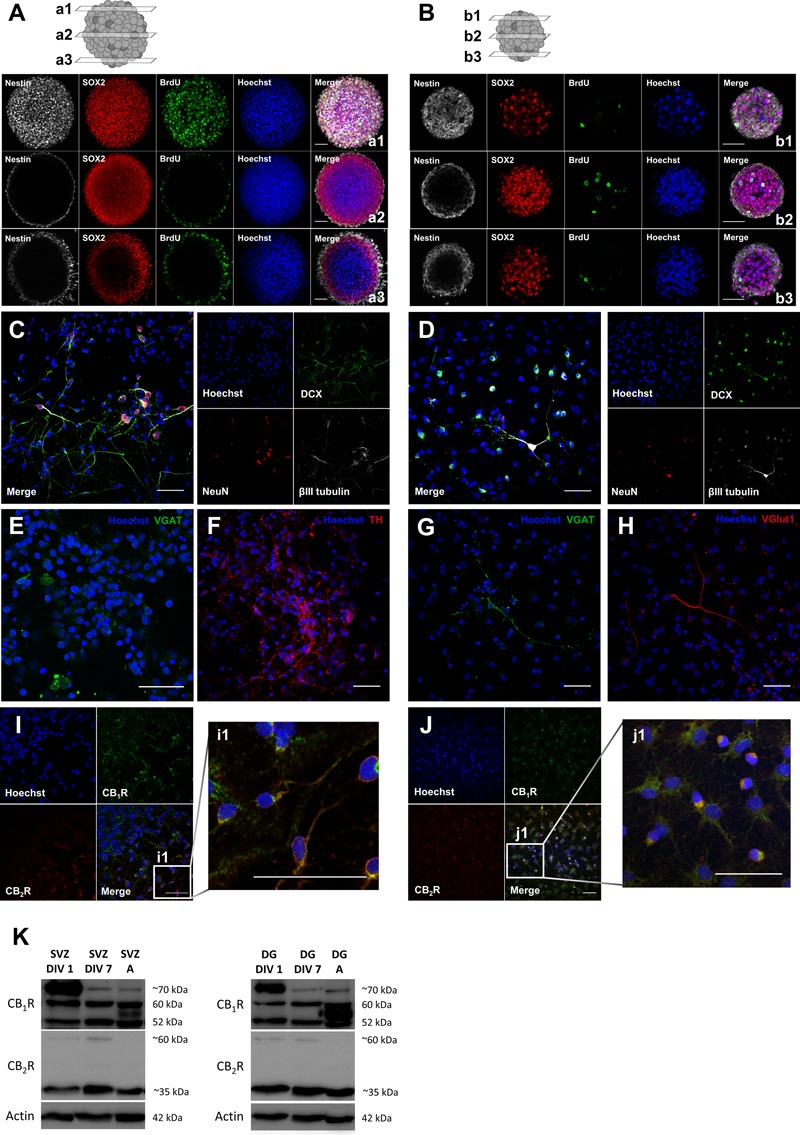
Characterization of SVZ and DG neurosphere cultures: neurospheres are composed by stem/progenitor cells and cells derived from neurosphere cultures undergo cell differentiation and express neuronal mature markers and both CB_1_R and CB_2_R. **(A,B)** Representative confocal digital images of a SVZ neurosphere **(A)** and a DG neurosphere **(B)** depicting Sox2 (red), BrdU (green), Nestin (white) and Hoechst 33342 (blue) immunoreactivity in 3 transverse planes (top, a/b1; middle, a/b2; bottom, a/b3). **(C–H)** Representative confocal digital images of SVZ cells **(C,E,F)** and DG cells **(D,G,H)** after 7 days of differentiation depicting immunoreactivity for immature and mature markers [DCX (green), NeuN (red), βIII tubulin (white) and Hoechst 33342 (blue)] **(C,D)** and for phenotypical markers [TH (red), VGlut (red), VGAT (green), and Hoechst 33342 (blue)] **(E–H)**. **(I,J)** Representative confocal digital images of SVZ cells **(I)** and DG cells **(J)** after 7 days of differentiation depicting CB_1_R (green), CB_2_R (red) and Hoechst 33342 (blue) immunoreactivity. **(K)** Detection of CB_1_R (molecular weight between 52 and ∼70 kDa) and CB_2_R (molecular weight of ∼35 and ∼60 kDa) by Western blotting in SVZ and DG cultures. For each immunoblot presented lane 1 corresponds to SVZ/DG proliferating cells (SVZ/DG DIV 1), lane 2 to SVZ/DG differentiated cells (SVZ/DG DIV 7) and lane 3 to SVZ/DG extracts from adult Sprague-Dawley rats (SVZ/DG A). Scale bars = 50 μm.

Using neurospheres as a method to study postnatal neurogenesis it was possible to observe that SVZ neurospheres are composed by stem/progenitor cells since they are formed by undifferentiated cells that express Sox2 (transcription factor important to maintain self-renewal) and Nestin (intermediate filament protein expressed by neuronal precursor cells) and, incorporate BrdU (**Figure 1A**). Importantly plating the neurospheres in differentiative conditions for 7 days (DIV 7) is sufficient to induce neuronal differentiation since a number of SVZ-derived cells express neuronal immature markers, such as doublecortin (DCX) and βIII tubulin and, also a marker for mature neurons, such as NeuN (**Figure 1C**). Furthermore, immunolabeling for the vesicular GABA transporter (VGAT, marker for GABAergic neurons) and the tyrosine hydroxylase (TH, marker for dopaminergic neurons) showed that SVZ-derived neurons express these proteins at DIV 7 (**Figures 1E,F**), which is in accordance with the fact that the two major phenotypes of SVZ-derived neurons are GABAergic and dopaminergic neurons. Similarly to SVZ cultures, DG neurospheres are also composed by undifferentiated cells expressing the stem/progenitor cell markers Sox2 and Nestin and with the ability to incorporate BrdU (**Figure 1B**). We have also observed that some DG-derived cells after 7 days in differentiative conditions (DIV 7) also express the immature markers DCX and βIII tubulin and the mature marker NeuN (**Figure 1D**). Additionally, DG-derived neurons at DIV 7 were found to express VGAT and vesicular glutamate transporter (VGlut1, marker for glutamatergic neurons), which reflects the two major phenotypes of DG-derived neurons, i.e., GABAergic and glutamatergic neurons (**Figures 1G,H**). Importantly, the expression of CB_1_R and CB_2_R in SVZ- and DG-derived cells was observed by immunocytochemistry and by western blotting (**Figures 1I–K**). In fact, CB_1_R and CB_2_R expression was found in both DIV 1 and DIV 7 SVZ and DG cells as well as in adult tissue (**Figure 1K**). In particular, a band for CB_1_R was found in the predicted molecular weight (52 kDa) and other bands at higher molecular weights (between 52 and ∼70 kDa) were also found, which probably correspond to glycosylated forms of the receptor (Song and Howlett, 1995; Bermúdez-Silva et al., 2008). Similarly, a band for CB_2_R was found at the predicted molecular weight (∼35 kDa) and other at ∼60 kDa which may also represent a glycosylated form (Gong et al., 2006; Bermúdez-Silva et al., 2008).

### CB_1_R Activation Stimulates SVZ Cell Proliferation

**FIGURE 2:**
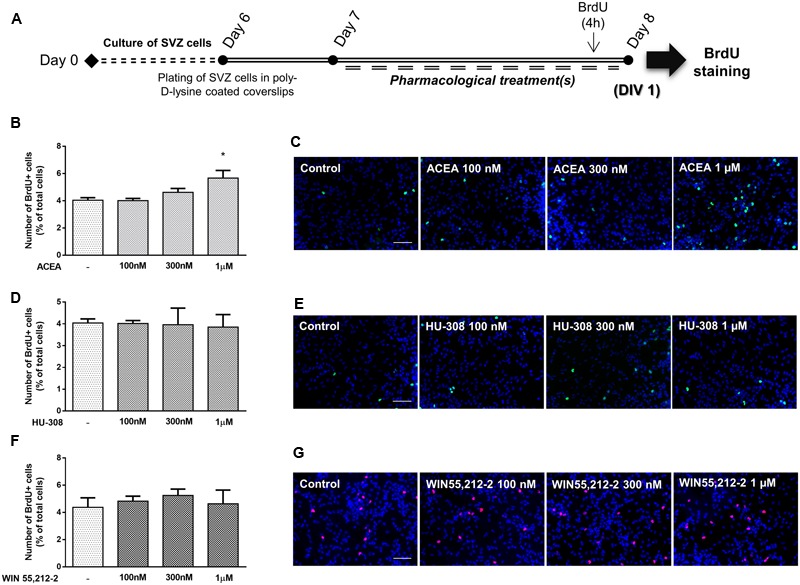
CB_1_R selective activation stimulates SVZ cell proliferation. **(A)** Schematic representation of the experimental protocol. Day 0 represents the day of cultures; at Day 6 SVZ neurospheres were plated for 24 h and at Day 7 cells were exposed to pharmacological treatments for further 24 h (Day 8). **(B,D,F)** Bar graphs depict the number of BrdU-positive cells expressed as percentage of total cells *per* culture. Data are expressed as mean ± SEM. *n* = 2–5. ^∗^*p* < 0.05 using Dunnett’s multiple comparison test. **(C,E,G)** Representative fluorescent digital images of cells immunopositive for BrdU (green for **C,E**; red for **G**) and Hoechst 33342 staining (blue nuclei) (in control cultures and in cultures exposed to ACEA, HU-308 and WIN55,212-2 at the indicated concentrations. Scale bars = 50 μm.

To determine if cannabinoid receptor activation modulates SVZ cell proliferation, increasing concentrations (100 nM, 300 nM, 1 μM) of selective CB_1_R (ACEA) and CB_2_R (HU-308) agonists and, non-selective CB_1_R and CB_2_R receptor agonist (WIN55,212-2) were applied on SVZ cells in SFM devoid of growth factors for 24 h. BrdU, a thymidine nucleotide analog, was added during the last 4 h of culture to label cells that went through S-phase. After fixation, incorporated BrdU was immunolabeled and positive nuclei were counted (**Figure 2A**). It was observed a significant increase in the number of BrdU-positive cells when cultures were incubated with 1 μM of the CB_1_R selective agonist ACEA, while 100 or 300 nM did not promote cell proliferation (control: 4.04 ± 0.18%; ACEA 1 μM: 5.67 ± 0.56%; *n* = 3, *p* < 0.05) (**Figures 2B,C**). No significant changes were observed in the number of BrdU-immunopositive nuclei obtained in cultures incubated with all the aforementioned concentrations of either the CB_2_R selective agonist HU-308 (**Figures 2D,E**) or the non-selective CB_1_R and CB_2_R agonist WIN55,212-2 compared with control cultures (**Figures 2F,G**). Therefore, concentrations of 1 μM were chosen based on the concentration-response curve.

**FIGURE 3:**
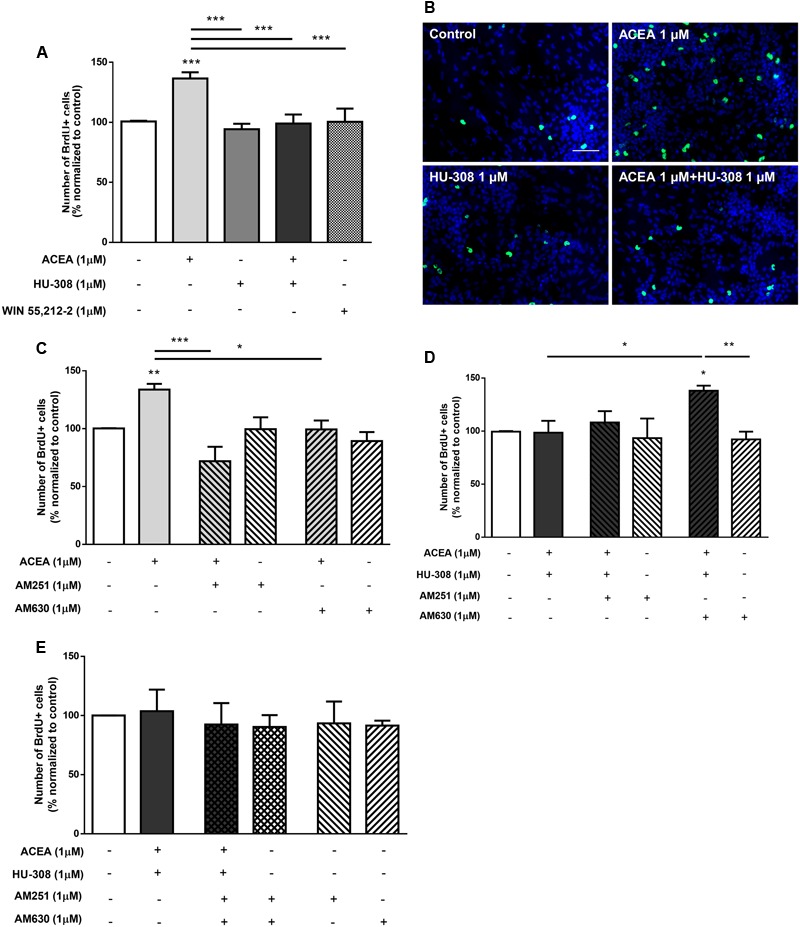
CB_1_R-mediated increase in SVZ cell proliferation is changed by modulation of CB_2_R. **(A,C–E)** Bar graphs depict the number of BrdU-positive cells. Values were normalized to the control mean for each experiment and are represented as mean ± SEM. Control was set to 100%. *n* = 3–11. ^∗^*p* < 0.05, ^∗∗^*p* < 0.01, ^∗∗∗^*p* < 0.001, using Sidak’s multiple comparison test. **(B)** Representative fluorescent digital images of cells immunopositive for BrdU (green) and Hoechst 33342 staining (bluenuclei). Scale bar = 50 μm.

We then investigated the interaction between CB_1_R and CB_2_R by combining selective activation of both CB_1_R and CB_2_R agonists. As previously described, it was detected a significant increase in the number of BrdU-positive cells when cultures were incubated with the CB_1_R selective agonist ACEA (control: 100.01 ± 0.15%; ACEA 1 μM: 136.50 ± 5.15%; *n* = 11, *p* < 0.001) compared with control cultures (**Figures 3A,B**). Concomitantly, no significant changes were obtained in cultures incubated with the CB_2_R selective agonist HU-308. Surprisingly when both CB_1_R and CB_2_R selective agonists were co-incubated the increase in cell proliferation mediated by CB_1_R activation was inhibited and therefore, had an effect similar to the observed by incubating with the non-selective CB_1_R and CB_2_R agonist WIN55,212 (**Figures 3A,B**). This suggests a role for CB_2_R in modulating the stimulatory effect promoted by CB_1_R on SVZ cell proliferation. To further elucidate the role of this CB_1_R-CB_2_R crosstalk on SVZ cell proliferation, cells were co-incubated with selective CB_1_R and CB_2_R selective antagonists (AM251 and AM630, respectively). The effect promoted by CB_1_R selective activation was blocked either in the presence of the CB_1_R selective antagonist AM251 (ACEA 1 μM + AM251 1 μM: 71.91 ± 12.49%; *n* = 3, *p* < 0.001) or in the presence of the CB_2_R selective antagonist AM630 (ACEA 1 μM + AM630 1 μM: 99.36 ± 7.76%; *n* = 5, *p* < 0.05) (**Figure 3C**), suggesting a putative interaction between CB_1_R and CB_2_R. Moreover, cells were further co-incubated with both CB_1_R and CB_2_R selective agonists (ACEA + HU-308) while being also incubated with either a CB_1_R or a CB_2_R antagonist, the data obtained being summarized in **Figure 3D**. When both selective agonists were present (ACEA + HU-308) and also in the presence of the CB_2_R selective antagonist, AM630, the effect promoted by ACEA alone on SVZ cell proliferation was rescued (ACEA 1 μM: 136.50 ± 5.15%; ACEA 1 μM+HU-308 1 μM + AM630 1 μM: 138.10 ± 4.77%; *n* = 5, *p* < 0.05). Incubation with the CB_1_R selective antagonist, AM251, prior to co-exposure with both selective agonists (ACEA + HU-308) had no effect. Moreover, when cells were co-incubated with both CB_1_R and CB_2_R selective agonists and antagonists no significant changes were observed in the number of BrdU-positive cells (control: 100.00 ± 0.05%; ACEA 1 μM + HU-308 1 μM + AM251 1 μM + AM630 1 μM: 92.40 ± 18.09%; *n* = 3) (**Figure 3E**). We also did not observe any significant alterations when incubating cultures with both selective antagonists alone (**Figures 3C–E**).

Taken together, the above results indicate that the effect observed on SVZ cell proliferation is via CB_1_R and that it is markedly affected while perturbing the degree of activation of CB_2_R. Overactivation of CB_2_R by exogenous agonists hampers the action of CB_1_R, but full blockade of CB_2_R in the absence of exogenous CB_2_R agonist also prevents the influence of CB_1_R upon SVZ cell proliferation.

### CB_1_R and CB_2_R Activation Induces SVZ Neuronal Differentiation

**FIGURE 4:**
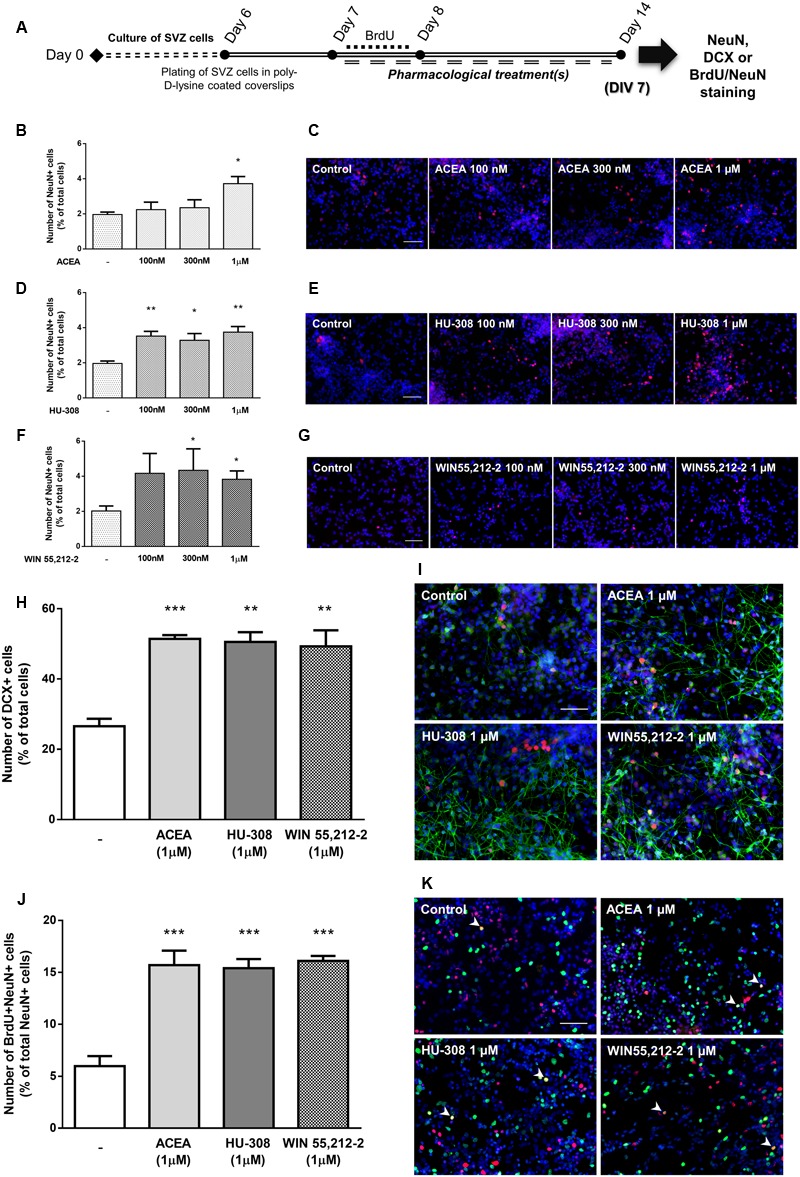
Selective and non-selective CBR activation induces SVZ neuronal differentiation. **(A)** Schematic representation of the experimental protocol. Day 0 represents the day of cultures; at Day 6 SVZ neurospheres were plated for 24 h and at Day 7 cells were exposed to pharmacological treatments for further 7 days (Day 14); for a subset of experiments BrdU was added for 24 h at Day 7. **(B,D,F,H,J)** Bar graphs depict the number of NeuN-positive cells expressed as percentage of total cells **(B,D,F)**; of DCX-positive cells expressed as percentage of total cells **(H)** and of BrdU/NeuN-positive cells expressed as percentage of total NeuN-positive cells **(J)** *per* culture. Data are expressed as mean ± SEM. *n* = 3–12. ^∗^*p* < 0.05, ^∗∗^*p* < 0.01, and ^∗∗∗^*p* < 0.001 using Dunnett’s multiple comparison test. **(C–K)** Representative fluorescent digital images of NeuN+ (red) cells **(C–G)**; DCX+ (green), NeuN+ (red) cells **(I)**; BrdU+ (green), NeuN+ (red) cells **(K)**. Hoechst 33342 staining in blue, arrowheads indicate BrdU/NeuN-positive cells. Scale bars = 50 μm.

To disclose whether cannabinoid receptor activation affects SVZ neuronal differentiation, SVZ cells were treated with increasing concentrations (100 nM, 300 nM, 1 μM) of selective CB_1_R (ACEA) and CB_2_R agonists (HU-308) and non-selective CB_1_R and CB_2_R agonist (WIN55,212-2) for 7 days in SFM without growth factor (**Figure 4A**). A significant increase in the number of NeuN-positive cells was observed when SVZ cultures were incubated with ACEA 1 μM but not with 100 or 300 nM, (control: 1.96 ± 0.14%; ACEA 1 μM: 3.72 ± 0.40%; *n* = 3–4, *p* < 0.05) (**Figures 4B,C**). When incubating SVZ cultures with HU-308, the CB_2_R selective agonist, we observed a significant increase in the number of NeuN-positive cells in all tested concentrations [control: 1.96 ± 0.14%; HU-308: 3.52 ± 0.27% (100 nM), 3.28 ± 0.38% (300 nM), 3.75 ± 0.32% (1 μM); *n* = 3–4, *p* < 0.05 and *p* < 0.01] (**Figures 4D,E**). Moreover, it was observed a marked increase in the number of NeuN-immunopositive nuclei when cells were incubated with increasing concentrations of WIN55,212-2, which reached statistical significance for concentrations ≥300 nM [control: 2.02 ± 0.28%; WIN55,212-2: 4.17 ± 1.13% (100 nM), 4.37 ± 1.23% (300 nM), 3.83 ± 0.47% (1 μM); *n* = 5–12, *p* < 0.05) (**Figures 4F,G**]. As with cell proliferation, 1 μM was the concentration selected for the following experiments. Interestingly, when we assessed whether selective or non-selective cannabinoid receptor activation would have an impact in the number of immature neurons, we observed a significant and robust increase in the number of DCX-positive cells with all receptor agonists used (control: 26.55 ± 2.12%; ACEA 1 μM: 51.43 ± 1.06%; HU-308 1 μM: 50.57 ± 2.75%; WIN55,212-2 1 μM: 49.30 ± 5.56%; *n* = 3, *p* < 0.01 and *p* < 0.001) (**Figures 4H,I**). To further support the role of cannabinoids in SVZ neuronal differentiation, we used a BrdU pulse for the first 24 h of treatments (CB_1_R and CB_2_R selective and non-selective activation) followed by a chase of 6 days (without BrdU) in the absence (control) or in the presence of the drug treatments (**Figure 4A**). We observed a 2.5-fold increase in the number of BrdU/NeuN-positive cells in all treated conditions (control: 5.97 ± 0.95%; ACEA 1 μM: 15.70 ± 1.38%; HU-308 1 μM: 15.40 ± 0.87%; WIN55,212-2 1 μM: 16.10 ± 0.49%; *n* = 4; *p* < 0.001), indicating that cannabinoid treatment is interfering in the early stages of SVZ neuronal differentiation, committing progenitors toward a neuronal fate (**Figures 4J,K**).

**FIGURE 5:**
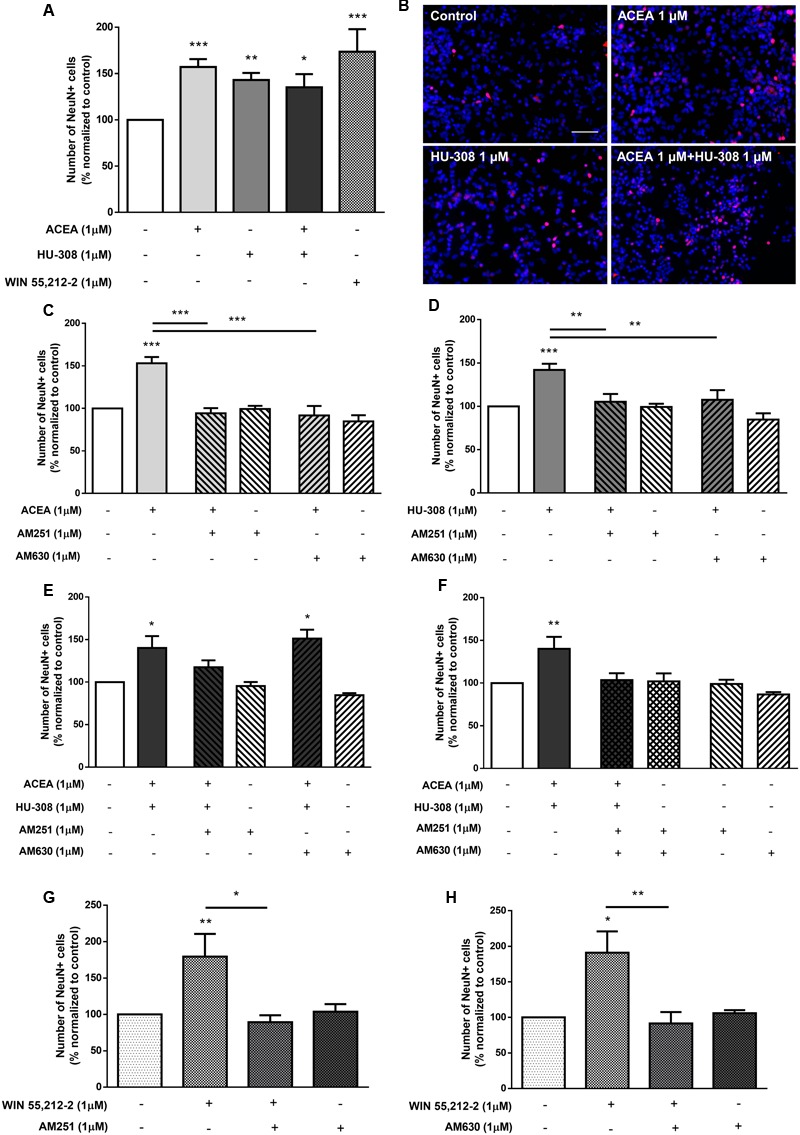
CB_1_R and CB_2_R tightly regulate SVZ neuronal differentiation. **(A,C–H)** Bar graphs depict the number of NeuN-positive cells. Values were normalized to the control mean for each experiment and are represented as mean ± SEM. Control was set to 100%. *n* = 3–15. ^∗^*p* < 0.05, ^∗∗^*p* < 0.01, ^∗∗∗^*p* < 0.001, using Sidak’s multiple comparison test. **(B)** Representative fluorescent digital images of cells immunopositive for NeuN (red) and Hoechst 33342 staining (blue). Scale bar = 50 μm.

To discriminate between the relative role of CB_1_R and CB_2_R upon neuronal differentiation, selective agonists for CB_1_R (ACEA, 1 μM) and for CB_2_R (HU-308, 1 μM) were used. Treatment of SVZ cells with either ACEA 1 μM or with HU-308 1 μM induced a significant (*p* < 0.001, *n* = 11–14) increase in the number of NeuN-positive cells (control: 99.99 ± 0.03%; ACEA 1 μM: 157.10 ± 8.50%; HU-308 1 μM: 143.00 ± 7.62%) (**Figures 5A,B**). The facilitatory effect upon neuronal differentiation was still evident when both agonists were added together (ACEA 1 μM + HU-308 1 μM: 135.20 ± 14.23%), but remarkably, the effect of both agonists was not additive, with a similar effect comparing with the non-selective CB_1_R and CB_2_R WIN55,212-2 (**Figures 5A,B**). The effect caused by CB_1_R selective activation was blocked either in the presence of the CB_1_R selective antagonist AM251 (ACEA 1 μM + AM251 1 μM: 94.18 ± 6.17%; *n* = 5–14, *p* < 0.001) or in the presence of the CB_2_R selective antagonist AM630 (ACEA 1 μM + AM630 1 μM: 91.74 ± 11.10%; *n* = 5, *p* < 0.001) (**Figure 5C**). Similarly, the effect caused by CB_2_R activation was blocked either in the presence of the CB_1_R selective antagonist AM251 (HU-308 1μM + AM251 1 μM: 105.20 ± 9.02%; *n* = 5–15, *p* < 0.01) or in the presence of the CB_2_R selective antagonist AM630 (HU-308 1 μM + AM630 1 μM: 105.20 ± 12.48%; *n* = 5, *p* < 0.01) (**Figure 5D**). This crossed-antagonism strongly suggests the existence of a CB_1_R-CB_2_R interaction to regulate SVZ neuronal differentiation. To further confirm the need of an interaction between CB_1_R and CB_2_R to modulate SVZ neuronal differentiation, co-incubation of both selective agonists and antagonists was performed, the results obtained being depicted in **Figures 5E,F**. Incubation of the CB_1_R selective antagonist, AM251, together with both selective receptor agonists (ACEA + HU-308) induced no significant changes in the number of NeuN-positive cells when compared to control conditions (control: 100.02 ± 0.67%; ACEA 1 μM + HU-308 1 μM + AM251 1 μM: 117.40 ± 8.13%; *n* = 3–12) (**Figure 5E**). Interestingly, incubation of the CB_2_R selective antagonist, AM630, with both selective agonists allowed an increase in the number of NeuN-positive cells (ACEA 1 μM + HU-308 1 μM + AM630 1 μM: 151.10 ± 10.47%; *n* = 6, *p* < 0.05) (**Figure 5E**), indicating that the influence of CB_1_R is preponderant over CB_2_R with respect to the modulation of SVZ neuronal differentiation. As expected, the effect promoted by both selective agonists, when added together, was abolished in the presence of both receptor antagonists (**Figure 5F**) (ACEA 1 μM + HU-308 1 μM + AM251 1 μM + AM630 1 μM: 103.50 ± 7.91%; *n* = 3–12). Additionally, no significant alterations were found when incubating cultures with both selective antagonists alone (**Figures 5C–F**). Interestingly, the effect promoted by non-selective activation of CB_1_R and CB_2_R with WIN55,212-2 on SVZ neuronal differentiation was blocked when cells were co-incubated with the CB_1_R selective antagonist AM251 (WIN55,212-2 1 μM + AM251 1 μM: 89.23 ± 9.60%) (**Figure 5G**) or with the CB_2_R selective antagonist AM630 (WIN55,212-2 1 μM + AM630 1 μM: 91.43 ± 16.07%) (**Figure 5H**), showing that the effect of WIN 55,212-2 on SVZ cell proliferation is dependent on both CB_1_R and CB_2_R.

**FIGURE 6:**
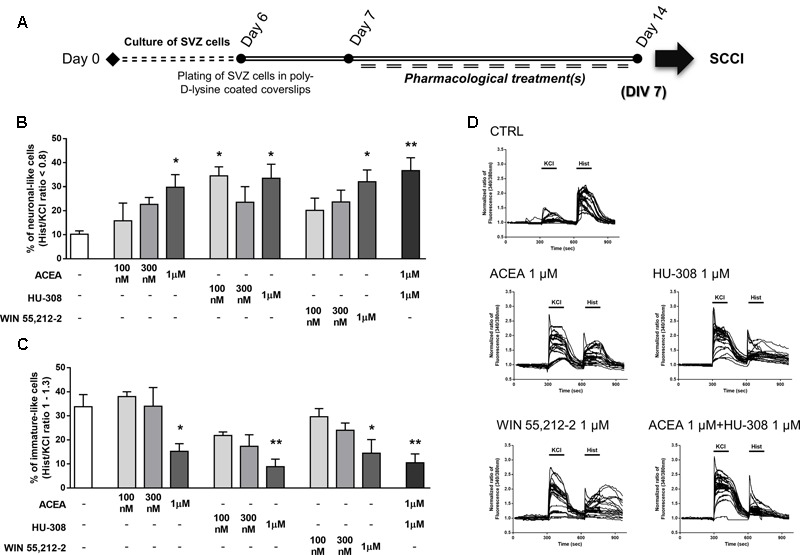
Selective and non-selective CBR activation promotes functional SVZ neuronal differentiation. **(A)** Schematic representation of the experimental protocol. Day 0 represents the day of cultures; at Day 6 SVZ neurospheres were plated for 24 h and at Day 7 cells were exposed to pharmacological treatments for further 7 days (Day 14). **(B,C)** Bar graphs depict the number of neuronal-like responding cells **(B)** and of immature-like responding cells **(C)** expressed as percentages of total cells analyzed by SCCI. Data are expressed as mean ± SEM. *n* = 3–4. ^∗^*p* < 0.05, ^∗∗^*p* < 0.01 using Dunnett’s multiple comparison test. **(D)** Representative SCCI profiles of response of 20 cells in control and ACEA, HU-308, WIN55,212-2, ACEA + HU-308 treated cultures.

In order to functionally evaluate neuronal differentiation of SVZ cells we used SCCI, as previously described (Xapelli et al., 2013). Briefly, variations of [Ca^2+^]_i_ at single cell level upon KCl and histamine (Hist) stimulations were measured and Hist/KCl ratios were calculated, therefore reflecting the phenotype of the analyzed cells. Ratios below 0.8 are characteristic of SVZ-derived neuronal-like cells whereas ratios between 1 and 1.3 are characteristic of SVZ-derived immature cells (Agasse et al., 2008). Quantification of the percentage of cells displaying a Hist/KCl ratio below 0.8 showed a concentration-dependent increase in the % of neuronal-like cells with the CB_1_R selective agonist ACEA [control: 10.21 ± 1.38%; ACEA: 15.83 ± 7.33% (100 nM), 22.67 ± 2.83% (300 nM), 29.75 ± 5.26% (1 μM); *n* = 3–4, *p* < 0.05], the CB_2_R selective agonist HU-308 [control: 10.21 ± 1.38%; HU-308: 34.50 ± 3.75% (100 nM), 23.50 ± 6.53% (300 nM), 33.50 ± 5.86 (1 μM); *n* = 3–4, *p* < 0.05] and with the CB_1_R and CB_2_R non-selective agonist WIN55,212-2 [control: 10.21 ± 1.38%; WIN55,212-2: 20.17 ± 5.05% (100 nM), 23.67 ± 4.88% (300 nM), 32.05 ± 4.93% (1 μM); *n* = 3–4, *p* < 0.05] (**Figures 6A,B**). Furthermore, selective activation of both receptors (ACEA + HU-308) also promoted a significant increase in the % of neuronal-like cells (ACEA 1 μM + HU-308 1 μM: 36.67 ± 5.40%; *n* = 3, *p* < 0.001) (**Figures 6A,B**). In fact, a three-fold increase in the number of neuronal-like cells resulting from incubation of these cannabinoids receptor agonists at concentrations of 1 μM was observed, indicating a robust cannabinoid-mediated augmentation of neuronal differentiation. Moreover, CB_1_R and/or CB_2_R selective and non-selective activation were also found to promote a significant decrease in the % of immature-like cells (ratio Hist/KCl 1–1.3) at 1 μM concentration (control: 33.80 ± 5.06%; ACEA 1 μM: 15.25 ± 3.19%; HU-308 1 μM: 8.87 ± 3.16%; WIN55,212-2 1 μM: 14.50 ± 5.65%; ACEA 1 μM+HU-308 1 μM: 10.50 ± 3.68%; *n* = 3–4, *p* < 0.05 and *p* < 0.01) (**Figures 6A,C**). While control cultures displayed a predominant immature-like profile, characterized by an increase in [Ca^2+^]_i_ in response to histamine but a small response or no response to KCl stimulation, the majority of ACEA-, HU-308-, WIN55,212-2-treated SVZ cultures displayed an increase in [Ca^2+^]_i_ in response to KCl but not to histamine stimulation which is consistent with a neuronal-like profile (**Figure 6D** and **Supplementary Figure S2**).

**FIGURE 7:**
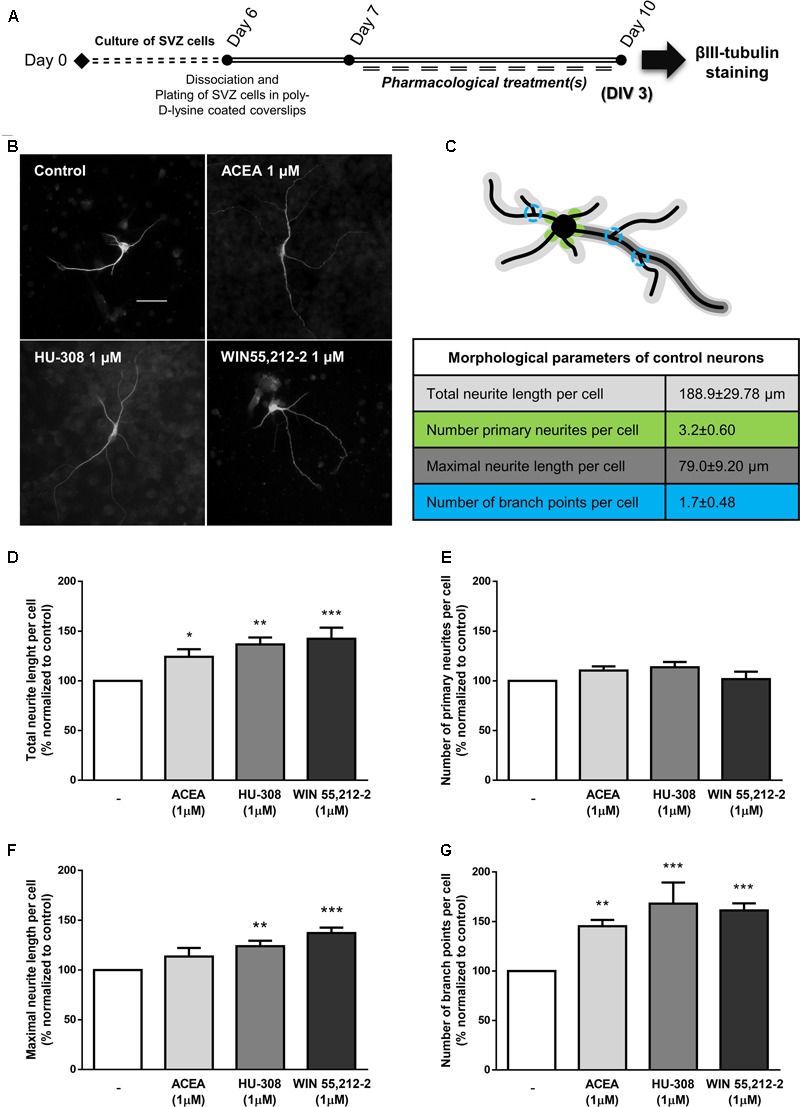
CB_1_R and CB_2_R activation induce neuronal maturation of SVZ cultures. **(A)** Schematic representation of the experimental protocol. Day 0 represents the day of cultures; at Day 6 SVZ neurospheres were dissociated and plated for 24 h and at Day 7 cells were exposed to pharmacological treatments for further 3 days (Day 10). **(B)** Representative digital images of βIII tubulin (white) positive cells in control and ACEA, HU-308, WIN55,212-2 treated cultures. **(C)** Illustrative image representing the color-coded morphological parameters evaluated and respective table with absolute values of control SVZ cultures for each parameter. **(D–G)** Bar graphs depict total length **(D)**, number of primary **(E)**, maximal length **(F)**, and number of ramifications **(G)** of neurites per cell. Values were normalized to the control mean for each experiment and are represented as mean ± SEM. Control was set to 100%. *n* = 4–9. ^∗^*p* < 0.05, ^∗∗^*p* < 0.01, ^∗∗∗^*p* < 0.001, using Sidak’s multiple comparison test. Scale bar = 30 μm.

Finally, in agreement with the observed proneurogenic action of cannabinoids, we investigated whether our treatments triggered neuronal morphological changes, consistent with neuronal maturation, by looking at neurite outgrowth and branching processes of neurons stained for βIII tubulin (**Figures 7A,B**). When comparing to control neurons (**Figure 7C**), incubation of SVZ cultures with ACEA, HU-308 and WIN55,212-2 induced a significant increase in the total length of neurites per neuronal cell (control: 100.0 ± 0.0%; ACEA 1 μM: 124.2 ± 7.60%; HU-308 1 μM: 136.7 ± 6.94%; WIN55,212-2 1 μM: 142.4 ± 11.13%; *n* = 4–9, *p* < 0.05, *p* < 0.01 and *p* < 0.001) (**Figure 7D**) and in the length of the maximal neurite (control: 100.0 ± 0.0%; ACEA 1 μM: 113.5 ± 8.66%; HU-308 1 μM: 124.0 ± 5.48%; WIN55,212-2 1 μM: 137.1 ± 5.47%; *n* = 4–9, *p* < 0.01 and *p* < 0.001) (**Figure 7F**). Furthermore, cannabinoid receptor agonists treatments promoted a marked increase in the number of branch points per cell when compared to control cultures (control: 100.0 ± 0.0%; ACEA 1 μM: 145.3 ± 6.32%; HU-308 1 μM: 168.1 ± 21.26%; WIN55,212-2 1 μM: 161.2 ± 7.22%; *n* = 4–9, *p* < 0.01 and *p* < 0.001) (**Figure 7G**) while not perturbing the number of primary neurites (**Figure 7E**).

### CB_1_R-CB_2_R Interaction Modulates DG Cell Proliferation

**FIGURE 8:**
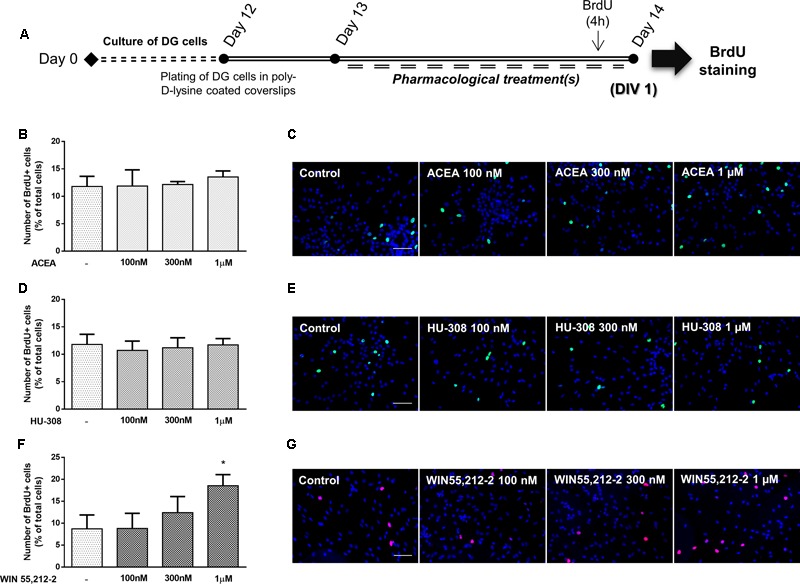
Non-selective CBR activation promotes DG cell proliferation. **(A)** Schematic representation of the experimental protocol. Day 0 represents the day of cultures; at Day 12 DG neurospheres were plated for 24 h and at Day 13 cells were exposed to pharmacological treatments for further 24 h (Day 14). **(B,D,F)** Bar graphs depict the number of BrdU-positive cells expressed as percentage of total cells *per* culture. Data are expressed as mean ± SEM. *n* = 2–5. ^∗^*p* < 0.05, using Dunnett’s multiple comparison test. **(C,E,G)** Representative fluorescent digital images of cells immunopositive for BrdU (green for **C,E**; red for **G**) and Hoechst 33342 staining (blue). Scale bars = 50 μm.

It was next investigated the effect of cannabinoid receptor activation on DG cell proliferation. For that purpose, DG cells were treated with increasing concentrations (100 nM, 300 nM, 1 μM) of CB_1_R and CB_2_R selective agonists and a non-selective CB_1_R and CB_2_R agonist for 24 h in SFM devoid of growth factors (**Figure 8A**). As shown in **Figures 8B–E**, CB_1_R selective activation with ACEA or CB_2_R selective activation with HU-308 did not promote any changes in the number of BrdU-positive cells in any tested concentration when compared with control conditions. Non-selective receptor activation with WIN55,212-2 caused a concentration-dependent increase in BrdU-positive cells, though the effect only reached statistical significance at 1 μM (control: 8.72 ± 3.16%; WIN55,212-2 1 μM: 18.55 ± 2.50%; *n* = 3–5, *p* < 0.05) (**Figures 8F,G**).

**FIGURE 9:**
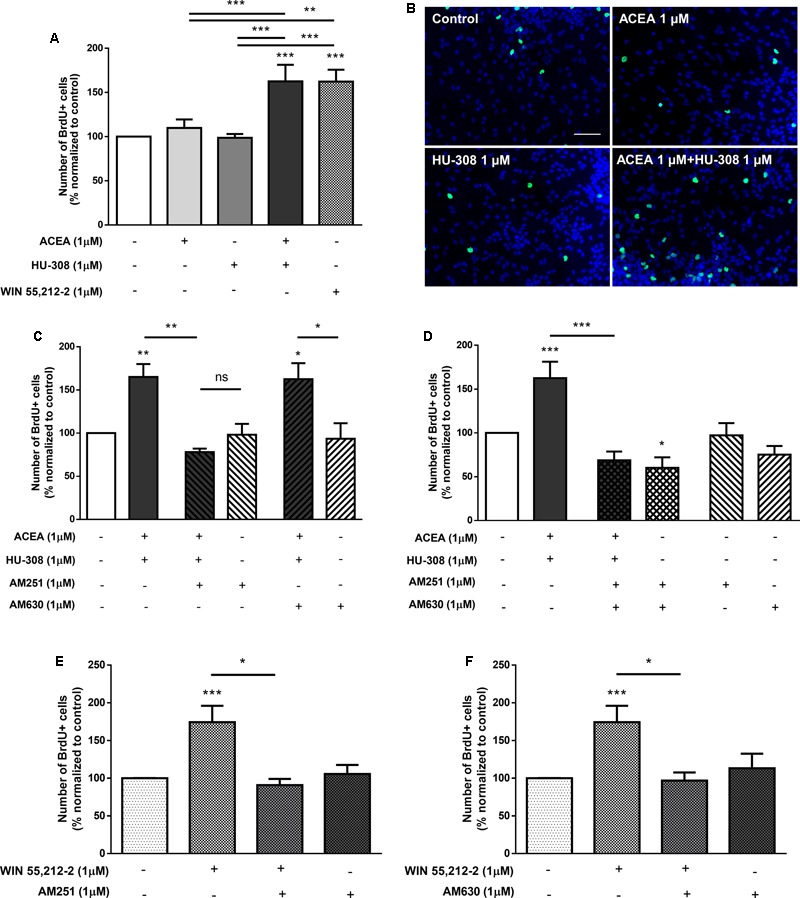
CB_1_R-CB_2_R interaction controls DG cell proliferation. **(A,C–F)** Bar graph depicts the number of BrdU-positive cells. Values were normalized to the control mean for each experiment and are represented as mean ± SEM. Control was set to 100%. *n* = 5–23. ^∗^*p* < 0.05, ^∗∗^*p* < 0.01 and ^∗∗∗^*p* < 0.001, using Sidak’s multiple comparison test. ns, non-significant. **(B)** Representative fluorescent digital images of cells immunopositive for BrdU (green) and Hoechst 33342 staining (blue). Scale bar = 50 μm.

To assess the relative role of each cannabinoid receptor, DG cultures were co-incubated with the selective receptor antagonists and agonists. There were no significant changes when incubating cultures with only ACEA or HU-308 (control: 100.00 ± 0.03%; ACEA 1 μM: 109.00 ± 9.65%; HU-308 1 μM: 98.68 ± 4.34%; *n* = 15, *p >* 0.05) (**Figures 9A,B**), when compared to control cultures, concomitant with the abovementioned results. However, when co-incubating cultures with both selective agonists for CB_1_R and CB_2_R (ACEA + HU-308) a surprisingly significant increase was observed in the number of BrdU-positive cells (ACEA 1 μM + HU-308 1 μM: 162.50 ± 18.83%; *n* = 10, *p* < 0.001), similar to the effect observed when cells were incubated with the non-selective receptor agonist WIN55,212-2 (**Figures 9A,B**). These results suggest that both receptors have to be active to induce DG proliferation. Furthermore, it was observed that in the presence of the CB_1_R selective antagonist, AM251, the effect promoted by incubation with both selective receptor agonists was lost (ACEA 1 μM + HU-308 1 μM + AM251 1 μM: 77.95 ± 4.12%; *n* = 5, *p* < 0.01) (**Figure 9C**), whereas co-incubation with the CB_2_R selective antagonist AM630 did not block the effect mediated by CB_1_R and CB_2_R co-activation (ACEA 1 μM + HU-308 1 μM + AM630 1 μM: 162.60 ± 18.44%; *n* = 5, *p* < 0.05) (**Figure 9C**). These data suggest that CB_1_R has a leading role in modulating DG proliferation. As could be expected, the effect promoted by co-incubation with both CB_1_R and CB_2_R selective agonists was completely lost when both receptor antagonists were present together (ACEA 1 μM + HU-308 1 μM + AM251 1 μM + AM630 1 μM: 68.76 ± 10.05%; *n* = 5, *p* < 0.01) (**Figure 9D**). Interestingly, when cultures were incubated with the CB_1_R and CB_2_R selective antagonists in the absence of agonists, a significant decrease on DG cell proliferation was observed (AM251 1 μM + AM630 1 μM: 60.15 ± 12.00%; *n* = 5, *p* < 0.05) (**Figure 9D**), suggesting a pivotal influence of endocannabinoids upon DG cell proliferation; single incubation with each of the receptor antagonist had no substantial effect (**Figures 9C,D**). Moreover, the increase on DG cell proliferation promoted by WIN55,212-2 1 μM was blocked either when cells were co-incubated with the CB_1_R selective antagonist AM251 (WIN55,212-2 1 μM+AM251 1 μM: 90.93 ± 8.10%; *n* = 5–23, *p* < 0.05) (**Figure 9E**) or with the CB_2_R selective antagonist AM630 (WIN55,212-2 1 μM + AM630 1 μM: 96.85 ± 10.86%; *n* = 5–23, *p* < 0.05) (**Figure 9F**), suggesting a CB_1_R-CB_2_R crosstalk mechanism mediating DG cell proliferation.

### DG Neuronal Differentiation Is Tightly Controlled by Cannabinoid Receptor Activation

**FIGURE 10:**
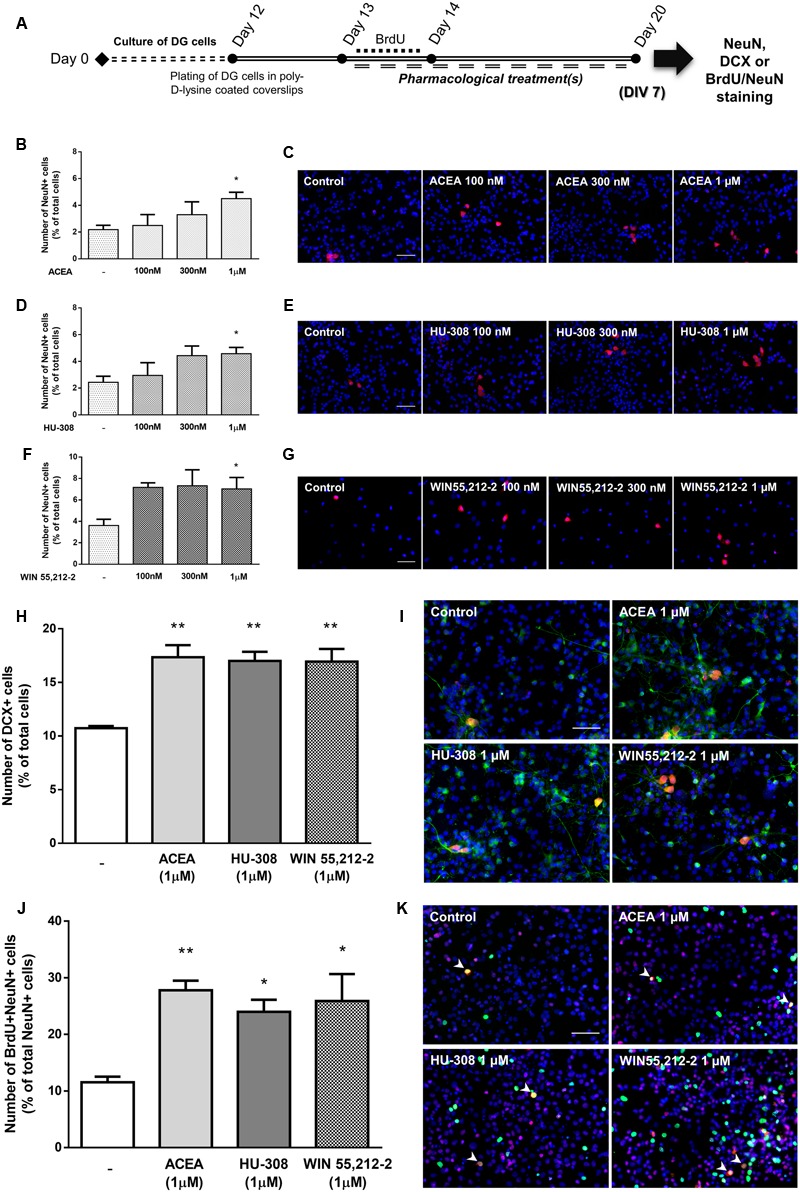
Selective and non-selective CBR activation induces DG neuronal differentiation. **(A)** Schematic representation of the experimental protocol. Day 0 represents the day of cultures; at Day 12 DG neurospheres were plated for 24 h and at Day 13 cells were exposed to pharmacological treatments for further 7 days (Day 20); for a subset of experiments BrdU was added for 24 h at Day 13. **(B,D,F,H,J)** Bar graphs depict the number of NeuN-positive cells expressed as percentage of total cells (B, D, F), of DCX-positive cells expressed as percentage of total cells **(H)** and of BrdU/NeuN-positive cells expressed as percentage of total NeuN-positive cells **(J)** *per* culture. Data are expressed as mean ± SEM. *n* = 3–10. ^∗^*p* < 0.05, ^∗∗^*p* < 0.01, using Dunnett’s multiple comparison test. **(C–K)** Representative fluorescent digital images of NeuN+ (red) cells **(C–G)**; DCX+ (green), NeuN+ (red) cells **(I)**; BrdU+ (green), NeuN+ (red) cells **(K)**. Hoechst 33342 staining in blue, arrowheads indicate BrdU/NeuN-positive cells. Scale bars = 50 μm.

To determine the effect of cannabinoid receptor activation on DG neuronal differentiation, DG cells were treated with increasing concentrations (100 nM, 300 nM, 1 μM) of CB_1_R and CB_2_R selective agonists and a non-selective CB_1_R and CB_2_R agonist in SFM devoid of growth factors for 7 days (**Figure 10A**). CB_1_R selective activation with ACEA promoted a concentration-dependent increase in the number of NeuN-positive cells, reaching statistical significance at the concentration of 1 μM (control: 2.18 ± 0.33%; ACEA 1 μM: 4.51 ± 0.47%; *n* = 3–6, *p* < 0.05) (**Figures 10B,C**). Similarly, when cultures were incubated with CB_2_R selective agonist HU-308 the same concentration-dependent profile was observed (control: 2.43 ± 0.44%; HU-308 1 μM: 4.57 ± 0.46%; *n* = 3–6, *p* < 0.05) (**Figures 10D,E**). An increase in the number of NeuN-positive cells was observed with all concentrations used for the non-selective receptor agonist WIN55,212-2, only reaching statistical significance at the concentration of 1 μM (control: 3.61 ± 0.58%; WIN55,212-2 1 μM: 7.03 ± 1.06%; *n* = 3–10, *p* < 0.05) (**Figures 10F,G**). When assessing the overall influence of cannabinoid receptors on early stages of DG neuronal differentiation by selectively activating CB_1_R (ACEA 1 μM) or CB_2_R (HU-308 1 μM) or, by non-selective receptor activation (WIN55,212-2 1 μM) we observed an increase in the number of immature neurons characterized by a significant increase in the number of DCX-positive cells (control: 10.73 ± 0.19%; ACEA 1 μM: 17.34 ± 1.13%; HU-308 1 μM: 17.00 ± 0.84%; WIN55,212-2 1 μM: 16.94 ± 1.18%; *n* = 3, *p* < 0.01) (**Figures 10H,I**), showing that activation of cannabinoid receptors functions as an early neurogenic propeller. Similarly to SVZ, we next investigated whether CB_1_R and CB_2_R activation could be committing DG early progenitors toward a neuronal fate. For that a BrdU pulse was done in the first 24 h of a 7-day treatment with ACEA, HU-308 and WIN55,212-2 (**Figure 10A**). We observed a substantial increase in the number of BrdU/NeuN-positive cells in all treated conditions (control: 11.55 ± 0.97%; ACEA 1 μM: 27.80 ± 1.69%; HU-308 1 μM: 23.99 ± 2.13%; WIN55,212-2 1 μM: 25.88 ± 4.76%; *n* = 3, *p* < 0.05 and *p* < 0.01) (**Figures 10J,K**), suggesting that CB_1_R and CB_2_R activation have an impact in the early stages of DG neuronal differentiation by inducing the commitment of early progenitors toward a neuronal fate.

**FIGURE 11:**
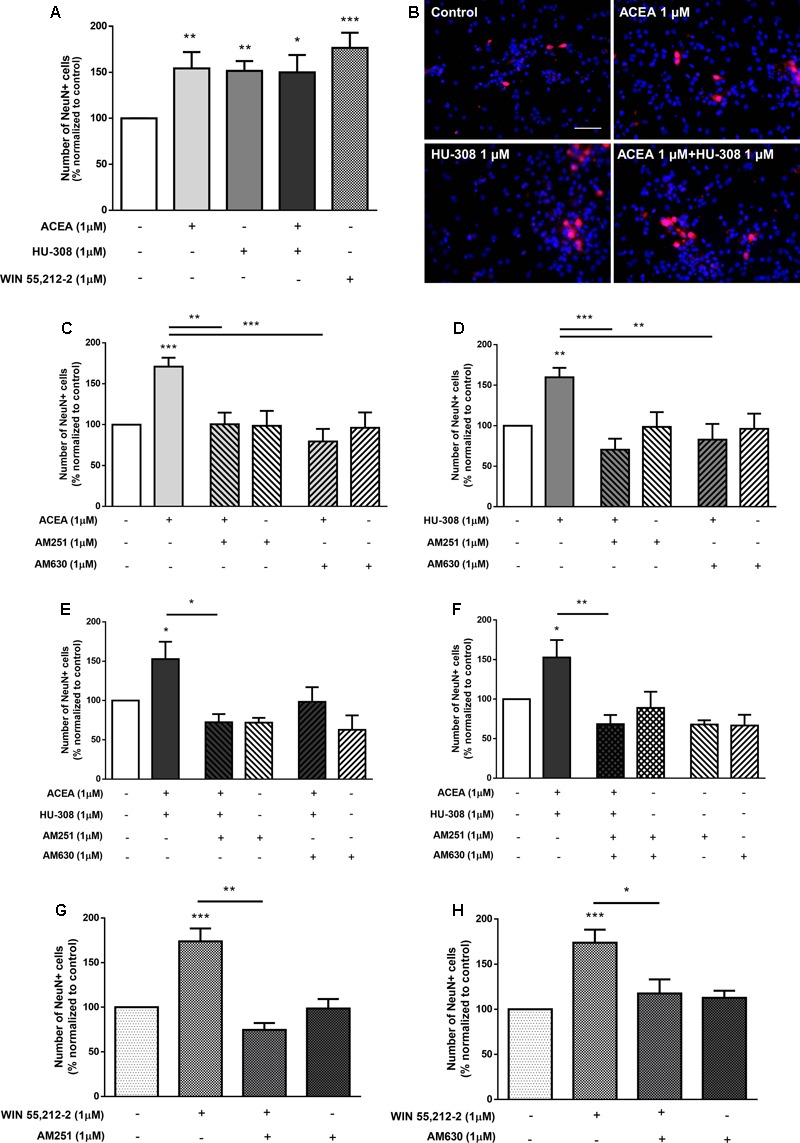
CB_1_R and CB_2_R crosstalk controls DG neuronal differentiation. **(A,C–H)** Bar graphs depict the number of NeuN-positive cells. Values were normalized to the control mean for each experiment and are represented as mean ± SEM. Control was set to 100%. *n* = 3–16. ^∗^*p* < 0.05, ^∗∗^*p* < 0.01, ^∗∗∗^*p* < 0.001, using Sidak’s multiple comparison test. **(B)** Representative fluorescent digital images of cells immunopositive for NeuN (red) and Hoechst 33342 staining (blue). Scale bar = 50 μm.

To evaluate whether the effect upon DG neuronal differentiation involves CB_1_R or CB_2_R, DG cells were treated with ACEA (1 μM) or HU-308 (1 μM) or a combination of both. It was observed a significant increase in the number of NeuN-positive cells when cultures were exposed to either the CB_1_R or the CB_2_R selective agonists (control: 100.02 ± 0.04%; ACEA 1 μM: 154.10 ± 17.78%; HU-308 1 μM: 151.50 ± 10.61%; *n* = 14–16, *p* < 0.01), as well as when both agonists were added together (ACEA 1 μM + HU-308 1 μM: 149.90 ± 18.81%; *n* = 7, *p* < 0.05) with an effect similar to the observed with the non-selective CB_1_R and CB_2_R agonist WIN55,212-2 (**Figures 11A,B**). Furthermore, the effect caused by CB_1_R selective activation was blocked either in the presence of the CB_1_R selective antagonist AM251 (ACEA 1 μM + AM251 1 μM: 100.50 ± 14.17%; *n* = 6, *p* < 0.01) or in the presence of the CB_2_R selective antagonist AM630 (ACEA 1 μM + AM630 1 μM: 79.42 ± 15.34%; *n* = 7, *p* < 0.001) (**Figure 11C**). Similarly, the effect caused by CB_2_R activation was blocked either in the presence of the CB_1_R selective antagonist AM251 (HU-308 1 μM + AM251 1 μM: 70.36 ± 13.58%; *n* = 7, *p* < 0.001) or in the presence of the CB_2_R selective antagonist AM630 (HU-308 1 μM + AM630 1 μM: 99.34 ± 22.89%; *n* = 6, *p* < 0.01) (**Figure 11D**). The CB_1_R selective antagonist AM251 alone was able prevent the effect of both agonists added together (ACEA + HU-308) (control: 100.04 ± 2.05%; ACEA 1 μM+HU-308 1 μM + AM251 1 μM: 72.26 ± 10.39%; *n* = 3, *p* < 0.05) (**Figure 11E**). Similarly, co-incubation with the CB_2_R selective antagonist AM630 blocked the effect mediated by CB_1_R and CB_2_R co-activation (ACEA 1 μM + HU-308 1 μM + AM630 1 μM: 98.35 ± 18.60%; *n* = 3) (**Figure 11E**). Additionally, when co-incubating with both selective antagonists, the effect promoted by both selective agonists was lost, compared to control cultures (control: 100.00 ± 0.43%; ACEA 1 μM + HU-308 1 μM + AM251 1 μM + AM630 1 μM: 68.36 ± 11.52%; *n* = 3, *p* < 0.01) (**Figure 11F**). In any case did the incubation with the antagonists alone cause significant effects as compared with control (**Figure 11**). Moreover, the effect promoted by 1 μM WIN55,212-2 on DG neuronal differentiation was blocked in the presence of the CB_1_R selective antagonist AM251 (WIN55,212-2 1 μM + AM251 1 μM: 74.61 ± 7.74%; *n* = 6, *p* < 0.01) (**Figure 11G**), as well as in the presence of the CB_2_R selective antagonist AM630 (WIN55,212-2 1 μM + AM630 1 μM: 117.20 ± 15.52%; *n* = 7, *p* < 0.05) (**Figure 11H**). Taken together, the above results point toward an action CB_1_R and CB_2_R as modulators of DG neuronal differentiation and further suggest the existence of crosstalk between CB_1_R and CB_2_R to control postnatal neurogenesis.

**FIGURE 12:**
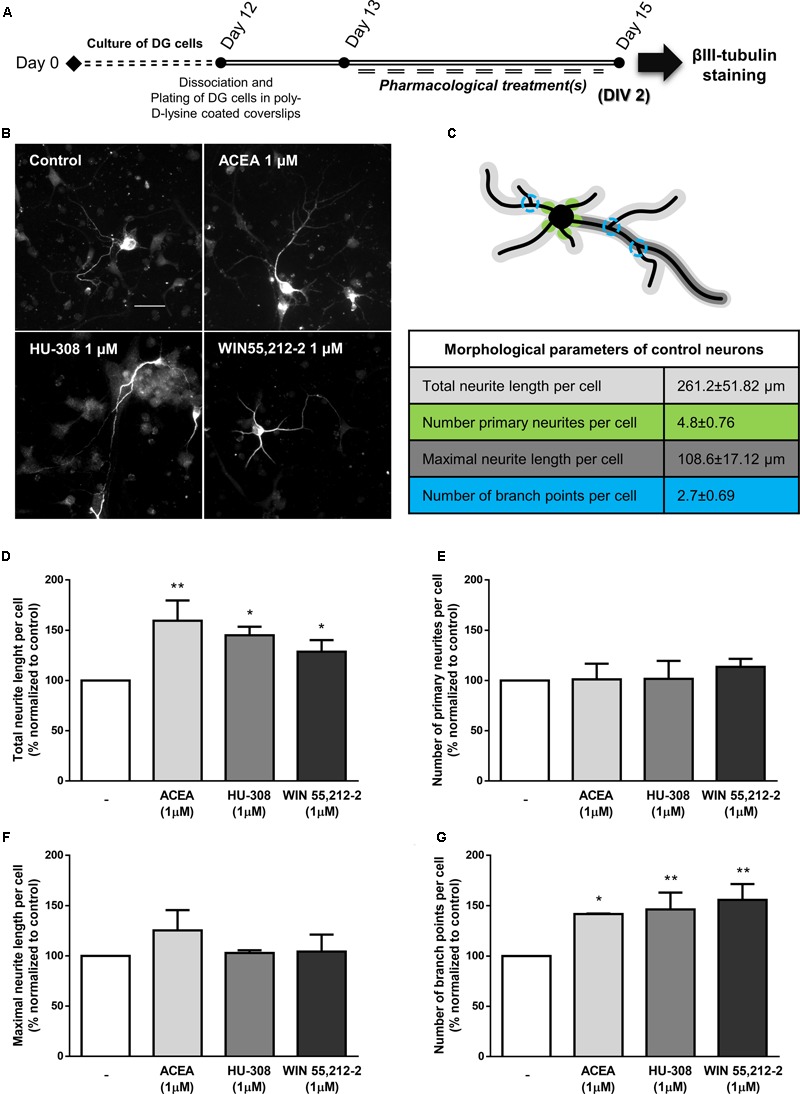
Activation of CBR induce neuronal maturation of DG cultures. **(A)** Schematic representation of the experimental protocol. Day 0 represents the day of cultures; at Day 12 DG neurospheres were dissociated and plated for 24 h and at Day 13 cells were exposed to pharmacological treatments for further 2 days (Day 15). **(B)** Representative digital images of βIII tubulin (white) positive cells in control and ACEA, HU-308, WIN55,212-2 treated cultures. **(C)** Illustrative image representing the color-coded morphological parameters evaluated and respective table with absolute values of control DG cultures for each parameter. **(D–G)** Bar graphs depict total length **(D)**, number of primary **(E)**, maximal length **(F)** and number of ramifications **(G)** of neurites per cell. Values were normalized to the control mean for each experiment and are represented as mean ± SEM. Control was set to 100%. *n* = 3-4. ^∗^*p* < 0.05, ^∗∗^*p* < 0.01, using Sidak’s multiple comparison test. Scale bar = 30 μm.

To further study the cannabinoid-stimulatory effect on DG neurogenesis, we treated DG cultures with selective and non-selective CB_1_R and CB_2_R agonists and evaluated the effect on neurite outgrowth of βIII tubulin-positive cells (**Figures 12A,B**). When comparing to control cultures (**Figure 12C**), CB_1_R and CB_2_R activation with the selective and non-selective receptor agonists promoted a significant increase in the total neurite length per cell (control: 100.0 ± 0.0%; ACEA 1 μM: 154.5 ± 20.15%; HU-308 1 μM: 145.0 ± 8.45%; WIN55,212-2 1 μM: 128.7 ± 11.51%; *n* = 3–4; *p* < 0.05 and *p* < 0.01) (**Figure 12D**). Moreover, although no changes were detected regarding the number of primary neurites per neuron and the maximal neurite length (**Figures 12E,F**) it was observed a marked increase in the number of branch points per cell when compared to control cultures (control: 100.0 ± 0.0%; ACEA 1 μM: 141.7 ± 0.48%; HU-308 1 μM: 146.3 ± 16.70%; WIN55,212-2 1 μM: 155.7 ± 15.81%; *n* = 3–4; *p* < 0.05 and *p* < 0.01) (**Figure 12G**). These results suggest that cannabinoid receptors are important modulators of DG neuronal maturation process.

### CB_1_ Receptors Co-immunoprecipitate with CB_2_ Receptors in SVZ and DG Cultures

**FIGURE 13:**
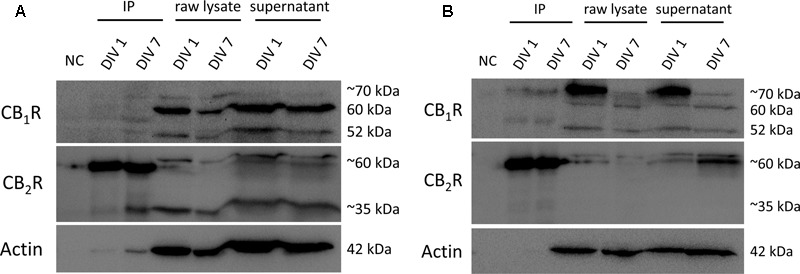
Co-immunoprecipitation (co-IP) of CB_1_R and CB_2_R. Co-immunoprecipitation assay to determine the interaction between CB_1_R (molecular weight between 52 and ∼70 kDa) and CB_2_R (molecular weight of ∼35 and ∼60 kDa) in SVZ cultures **(A)** and in DG cultures **(B)** at both DIV 1 and DIV 7. NC, negative control (beads + sample without antibody); IP, sample bound to antibody-bead mixture; raw lysate, lysed SVZ and DG cultures; supernantant, sample not bound to antibody-bead mixture.

The overall crosstalk actions of CB_1_R and CB_2_R in SVZ and DG cultures hints at a possible physical interaction of these receptors. To test this hypothesis, we performed immunoprecipitation with an anti-CB_2_R receptor antibody in SVZ and DG cultures at DIV 1 and DIV 7 (molecular weight of ∼35 and 60 kDa). Subsequent immunoblot analyses of the composition of the immunoprecipitates showed a tenuous detection of CB_1_R at ∼52 and 60 kDa molecular weight in SVZ (**Figure 13A**) and DG (**Figure 13B**) cultures at both time points suggesting that CB_1_R and CB_2_R interact at the protein–protein physical level.

## Discussion

A main finding herein reported for the first time is the crosstalk between cannabinoid CB_1_ and CB_2_ receptors in the control of proliferation and differentiation of SVZ- and DG-derived NSPC. It was observed that CB_1_R and CB_2_R co-activation had no additive effect when compared with the effect mediated by the receptor agonists alone. Importantly, selective activation of CB_1_R induced an increase in SVZ cell proliferation that was lost when CB_1_R and CB_2_R were co-activated. Surprisingly, regarding DG cell proliferation, the selective activation of CB_1_R or CB_2_R has no effect but co-incubation with both CB_1_R and CB_2_R agonists promoted an increase in proliferation similar to the observed with a CB_1_R and CB_2_R non-selective agonist, indicating the need of simultaneous activation of the two receptors to facilitate DG cell proliferation, in clear contrast with the SVZ cell proliferation, where the CB_1_R is the predominant receptor to control proliferation. A constant finding in both neurogenic niches and in the modulation of either proliferation or differentiation, is the cross-antagonism between CB_1_R and CB_2_R (**Table 3**).

**Table 3:** Summary table.

*Treatments*	SVZ cells	DG cells
**Cell proliferation**		
Non-selective CBR activation	**=**	**↑ ↑ ↑**
CB_1_R activation	**↑ ↑ ↑**	**=**
CB_2_R activation	**=**	**=**
CB_1_R + CB_2_R activation	**=**	**↑ ↑ ↑**
CB_1_R blockage	**–]** CB_1_R effect	**–]** CB_1_R + CB_2_R effect
CB_2_R blockage	**–]** CB_1_R effect	**–]** CB_1_R + CB_2_R effect
**Neuronal differentiation**		
Non-selective CBR activation	**↑ ↑ ↑**	**↑ ↑ ↑**
CB_1_R activation	**↑ ↑ ↑**	**↑ ↑ ↑**
CB_2_R activation	**↑ ↑ ↑**	**↑ ↑ ↑**
CB_1_R + CB_2_R activation	**↑ ↑ ↑**	**↑ ↑ ↑**
CB_1_R blockage	**–]** Non-selective CBR effect **–]** CB_1_R/CB_2_R effect	**–]** Non-selective CBR effect **–]** CB_1_R/CB_2_R effect **–]** CB_1_R + CB_2_R effect
CB_2_R blockage	**–]** Non-selective CBR effect **–]** CB_1_R / CB_2_R effect 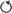 CB_1_R+CB_2_R effect	**–]** Non-selective CBR effect**–]** CB_1_R/CB_2_R effect
**Neuronal maturation**		
Non-selective CBR activation	**↑ ↑ ↑**	**↑ ↑ ↑**
CB_1_R activation	**↑ ↑ ↑**	**↑ ↑ ↑**
CB_2_R activation	**↑ ↑ ↑**	**↑ ↑ ↑**

***↑ ↑ ↑** significant increase; = no significant effect; –] effect blockage; 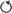 restored effect.*

Firstly, we detected by western blotting and immunocytochemistry that cells derived from SVZ and DG rat neurospheres express CB_1_R and CB_2_R. This is in accordance with several authors which have shown that both CB_1_R and CB_2_R are expressed in these regions (Jin et al., 2004; Aguado et al., 2005).

Interestingly we observed that HU-308 (CB_2_R selective agonist) and WIN55,212-2 (CB_1_R and CB_2_R non-selective agonist) treatments had no effect on SVZ cell proliferation. Nevertheless, selective CB_1_R activation with ACEA promoted a significant increase in cell proliferation of SVZ cultures, an effect that was lost when cultures were co-incubated together with CB_1_R or CB_2_R selective antagonists. Several reports showed that CB_1_R activation indeed promoted SVZ cell proliferation (Trazzi et al., 2010; Díaz-Alonso et al., 2012; Compagnucci et al., 2013; Xapelli et al., 2013). Moreover, CB_1_R and CB_2_R activation was found to promote proliferation of primary murine cortical neurospheres (Rubio-Araiz et al., 2008) and of mouse NSPC via IL-1 signaling pathways (García-Ovejero et al., 2013). Others have also shown that CB_2_R activation could promote proliferation in embryonic cell lines, in SVZ neurosphere cultures and in the SVZ of young mice (Goncalves et al., 2008; Palazuelos et al., 2012). However, in our experimental conditions, CB_2_R activation did not induce cell proliferation. Interestingly, the observed increase in SVZ cell proliferation mediated by CB_1_R selective activation was abolished in the presence of the CB_2_R selective agonist. In fact, this result may help to explain why treatment with the non-selective CB_1_R and CB_2_R agonist had no effect. Importantly, the effect promoted by activation of CB_1_R in cell proliferation was reestablished in SVZ cells incubated with the selective agonists for CB_1_R and CB_2_R that were pre-treated with the CB_2_R selective antagonist. These findings suggest a negative crosstalk between CB_1_R and CB_2_R.

Regarding SVZ neuronal differentiation, it was found to be significantly increased upon WIN55,212-2 treatment as well as with CB_1_R and/or CB_2_R selective activation. The effect promoted by WIN55,212-2 was lost when cultures were co-incubated with either CB_1_R or CB_2_R selective antagonists. Similarly, the effect mediated by CB_1_R or CB_2_R in SVZ neuronal differentiation was blocked in the presence of CB_1_R or CB_2_R antagonists. Selective and non-selective cannabinoid receptor activation was shown to stimulate SVZ neuronal differentiation not only by promoting an increase in the number of NeuN-positive cells but also by expanding the pool of immature neurons, i.e., by increasing the number of DCX-positive cells and by accelerating the differentiation of proliferating neuronal precursor cells, i.e., by increasing the number of BrdU/NeuN-positive cells. In fact, we showed by SCCI that CB_1_R and CB_2_R activation induces functional neuronal differentiation in SVZ cell cultures, reflected by a striking three-fold increase in the number of neuronal-like cells. Moreover, treatment with CB_1_R and/or CB_2_R agonists enhanced neuronal maturation by promoting a more pronounced arborization of newly formed neurons, being observed a higher number of ramifications and an increase in neurite length. Throughout literature, the existing data appears to be conflicting. In fact, the work done by Compagnucci et al. (2013) and Xapelli et al. (2013) also shows that CB_1_R activation is important to promote neuronal differentiation. However, Aguado et al. (2006) and Gomez et al. (2010) showed that endocannabinoid treatment promoted astroglial and/or oligodendroglial differentiation rather than neuronal differentiation, while adult CB_1_R knockout resulted in a decrease in neural proliferation (Aguado et al., 2006). Our data strongly suggests a pivotal two-part interaction between CB_1_R and CB_2_R in the regulation of SVZ proliferation and neuronal differentiation and maturation, highlighting the need to interpret results that involve the prevention of the activation of one receptor on the light of its impact upon the other receptor subtype, thus helping to understand some inconsistencies reported in the literature.

While SVZ neurogenesis might be particularly relevant in olfactory control as well as in the putative inhibition upon neurodegeneration (Curtis et al., 2007), the role of cannabinoids on hippocampal neurogenesis has been intensively studied in an attempt to understand how endocannabinoids can shape learning and memory processes. Evidence so far observed show that the number of proliferating cells in the DG is reduced in CB_1_R- or CB_2_R-deficient mice and that CB_1_R and CB_2_R play major roles in NSPC proliferation, morphogenesis and differentiation (Palazuelos et al., 2006; Molina-Holgado et al., 2007; Galve-Roperh et al., 2013). Therefore, we explored whether CB_1_R-CB_2_R crosstalk could have an impact on DG cell proliferation and neuronal differentiation. We observed that treatment with CB_1_R and CB_2_R non-selective agonist promoted an increase in DG cell proliferation and that this effect was lost when cultures were co-treated with the CB_1_R or CB_2_R selective antagonists. Surprisingly, no changes were observed when cultures were treated with CB_1_R or CB_2_R selective agonists alone. Remarkably, co-incubation of both selective CB_1_R and CB_2_R agonists resulted in a significant increase in DG cell proliferation. Together, these results strongly indicate the need of co-activation of both CB_1_R and CB_2_R to affect DG proliferation. The effect promoted by co-incubation with the CB_1_R and CB_2_R agonists was blocked in the presence of the CB_1_R antagonist, but not by the CB_2_R antagonist, suggesting that CB_1_R plays a major role in this process. The current available data about the effects promoted by CB_1_R or CB_2_R on DG cell proliferation is still quite controversial. In fact, some reports demonstrated that DG cell proliferation and survival was significantly impaired when using CB_1_R and CB_2_R knockout mice models (Aguado et al., 2006; Palazuelos et al., 2006) and that CB_1_R or CB_2_R activation *per se* promoted an increase in cell proliferation in the DG (Jiang et al., 2005; Palazuelos et al., 2012) or rescued the deleterious effect on NSPC proliferation promoted by ethanol (Rivera et al., 2015). However, other studies showed that CB_1_R antagonism resulted in an enhanced DG cell proliferation (Hill et al., 2006; Wolf et al., 2010). Our data is in accordance with Aguado et al. (2005) that observed that WIN55,212-2 treatment promoted an increase on DG cell proliferation and advances a step in the field by revealing a tight interaction between CB_1_R and CB_2_R in the regulation of this process.

Treatment with the CB_1_R and CB_2_R non-selective agonist also stimulated DG neuronal differentiation. Moreover, the same effect was observed when DG cells were treated with either CB_1_R or CB_2_R selective agonists. In the case of neuronal differentiation, data indicates that activation of only one or the two receptors was enough to trigger this effect. These results are in accordance with the work of Compagnucci et al. (2013) in which AEA enhances cell differentiation toward a neuronal lineage, via a CB_1_R-dependent mechanism. Furthermore, the work by Wolf et al. (2010) also showed that in the absence of CB_1_R, neuronal differentiation is reduced. Additionally, it was also showed that administration of a CB_2_R agonist promoted differentiation of human NSPC (Avraham et al., 2014). In our experimental conditions, CB_1_R and/or CB_2_R activation induced DG neuronal differentiation, being observed both a marked increase in the number of immature neurons (DCX-positive cells) and a substantial increase in the number of progenitor cells labeled with BrdU that ultimately differentiated into NeuN-positive mature neurons (BrdU/NeuN-positive cells). Furthermore, treatment with CB_1_R and/or CB_2_R agonists promoted a potentiation of DG neuronal maturation processes, i.e., neurite length and branching. Importantly, the novelty of our data resides in the observation that the effect mediated by the CB_1_R selective agonist on neuronal differentiation was blocked with CB_1_R or CB_2_R antagonists and the same was observed regarding the effect promoted by the CB_2_R selective agonist. Likewise, WIN55,212-2 (CB_1_R and CB_2_R non-selective agonist)-mediated increase in DG neuronal differentiation was blocked in the presence of CB_1_R or CB_2_R selective antagonists. Hence, these data demonstrates that, though both receptors can independently affect DG neuronal differentiation, they do so in a close interaction so that, by blocking only one of them, the CB_1_R-CB_2_R-mediated effect is lost. This phenomenon of cross-antagonism has been considered a fingerprint of heteromerization, thus expression of CB_1_R-CB_2_R heteromers may explain how CB_1_R and CB_2_R control DG neuronal differentiation.

Hence, to address this issue, we performed co-IP experiments using DIV 1 and DIV 7 SVZ and DG cultures. The co-IP data suggest CB_1_R to be associated with CB_2_R, indicating the possible existence of CB_1_R-CB_2_R heteromers in our experimental conditions.

Divergent effects are observed throughout literature concerning the role of endocannabinoids on neurogenesis which may be partly explained from the use of different pharmacological approaches or differences in study design, animal species or gender used. From our study it becomes clear that the use of non-selective cannabinoid receptor ligands may account for the differences among studies. Furthermore, as observed by Jin et al. (2004), treatment with SR141716A, a CB_1_R antagonist, promoted an increase in cell proliferation in the SVZ of CB_1_R-KO animals, showing that this effect is not dependent of CB_1_R. The age of the animal is also a determining factor. In fact, Abboussi et al. (2014) have showed that chronic administration of WIN55,212-2 to rats during adulthood had no effect on the number of immature neurons in the DG; however, administration during adolescence decreased the number of immature neurons. Using the same compound, other group found that a low, continuous administration significantly increased neurogenesis in aged rats (Marchalant et al., 2009). Moreover, Aguado et al. (2005), through an *in vitro* approach, also showed that WIN55,212-2 promoted an increase in the number of neural progenitors and generation of neurospheres. These findings demonstrate that compound selectivity and the system that it is used on are extremely important factors when studying neurogenesis, and that regulation of this process may happen at a multidimensional level, which accounts for the complexity/variety of the system.

Overall, our results show that both proliferation and differentiation/maturation are being stimulated upon pharmacological cannabinoid receptor activation. In fact, cannabinoid receptors are important modulators of neurogenesis by acting at distinct neurogenic phases (Aguado et al., 2005; Xapelli et al., 2013; Avraham et al., 2014; Prenderville et al., 2015). Moreover, our results suggest that activation of CB_1_R and/or CB_2_R, although being important for cell proliferation, plays a preponderant role in stimulating neuronal differentiation and maturation processes in both SVZ and DG cells. Our data also shows that SVZ and DG neurogenic niches respond differently to the same pharmacological treatments. Particularly regarding cell proliferation, WIN55,212-2 treatment was found to promote DG cell proliferation but not SVZ cell proliferation, whereas CB_1_R selective activation with ACEA promoted SVZ cell proliferation and only co-activation of CB_1_R and CB_2_R induced DG cell proliferation (**Table 3**). These differences can be to some extent due to the properties of each NSPC population and the heterogeneity within each NSPC pool (Göritz and Frisén, 2012). In fact, there are divergences between the two niche microenvironments regarding molecules regulating morphogenesis, rates of division and survival which may account for the observed differences (Curtis et al., 2012; Bonfanti, 2014).

Notably, part of the pharmacological differences could be explained by the existence of CB_1_R-CB_2_R heteromers. A heteromer receptor is, by definition, a macromolecular complex composed of at least two functional receptor units with biochemical properties that are demonstrably different from those of its individual receptors (Ferré et al., 2009a). BRET and PLA approaches have indeed shown that CB_1_R and CB_2_R co-localize in HEK-293T cells and SH-SY5Y neuroblastoma cells as well as puts into evidence the presence of CB_1_R-CB_2_R heteromers in a variety of rat brain regions (pineal gland, nucleus accumbens and globus pallidus) (Callén et al., 2012). Furthermore, in the same study, the authors observed a functional interdependence between CB_1_R and CB_2_R that varyingly modulated Akt/PKB signaling and neurite outgrowth. Moreover, an *in situ* assay showed the presence of CB_1_R-CB_2_R heteromers within basal ganglia output neurons in macaques (Sierra et al., 2015). In the same way, it was already shown that CB_1_Rs and CB_2_Rs are expressed in rat SVZ and DG tissue and neurosphere-derived cells (Morozov and Freund, 2003; Gong et al., 2006; Arévalo-Martín et al., 2007). Particularly, NSPC express a functional endocannabinoid system and are able to produce both AEA and 2-AG and, also, that are targeted by cannabinoids to promote neurosphere generation and NSPC proliferation (Aguado et al., 2005; Palazuelos et al., 2006). Similarly to the study of Callén et al. (2012), our study also reveals a bidirectional cross-antagonism in which CB_1_R antagonists have the ability to block CB_2_R agonist-mediated effects on neurogenesis and vice-versa, a phenomenon that occurs in either SVZ- or DG-derived NSPC. Importantly, our study expands the evidence of the relevance of CB_1_-CB_2_ receptor crosstalk in neuronal differentiation and proliferation. It is likely that differences between the SVZ and DG niches may result from different expression of CB_1_R-CB_2_R heteromers that, eventually, translates into differences in the proliferation or differentiation rates of each niche cell population.

While much progress has been made in recent decades to understand the role of endocannabinoids on neurogenesis, our results provide a new perspective on how CB_1_R and CB_2_R might be modulating each other to control neurogenesis putatively through CB_1_R-CB_2_R heteromer complexes. One explanation for the mechanism behind this interaction may be that direct antagonism toward one receptor may be blocking the function of the entire heteromer. Also, similarly to the opioid receptor pharmacology, in which heteromerization between the μ-opioid receptor and δ-opioid receptor shows a “biased antagonism” (Milan-Lobo and Whistler, 2011), one receptor can be using the other as an antagonizing signal (or vice-versa) when both CB_1_R and CB_2_R are co-expressed in the same NSPC, having a significant impact on receptor signaling and, ultimately, in the regulation of neurogenesis. In other cases, both receptors may need to be co-activated in a concerted way so that correct signaling between them occurs. Indeed, this interdependent CB_1_R-CB_2_R modulation of neurogenesis may be occurring at distinct levels of action or at unique temporal time-windows resulting in different pivotal roles depending on the region NSPC are obtained, that is SVZ or DG, or on which neurogenic stage NSPC are in.

Taken together, our study demonstrates a clear CB_1_R-CB_2_R interaction that is responsible for a differential modulation of SVZ and DG neurogenic properties, such as proliferation, neuronal differentiation and maturation. Further studies are required to both define the importance of this crosstalk and elucidate the mechanisms involved, that may result either from protein–protein interaction (formation of heteromeric receptor complexes), mediated by G proteins, effectors, or second messengers. Certainly, future high-resolution imaging studies will be required to determine the dynamics of this receptor-receptor interaction within NSPC derived from both neurogenic niches and its contribution to proliferation and differentiation processes. For that reason, the designing of cannabinoid-based pharmacological tools that may prove promising must take into account the existence of this complex heteroreceptor crosstalk. Targeting cannabinoid receptors in a combined manner rather than each one individually may thus prove itself to be more efficient when designing therapeutical approaches to enhance neurogenesis.

## Author Contributions

RR: conception and design of the work, acquisition, analysis and interpretation of data for the work; drafting and revising critically the work for important intellectual content and final approval of the version to be published. FR: conception and design of the work, acquisition, analysis and interpretation of data for the work; revising critically the work for important intellectual content and final approval of the version to be published. FF: acquisition, analysis and interpretation of data for the work; revising critically the work for important intellectual content and final approval of the version to be published. SV: acquisition, analysis and interpretation of data for the work; revising critically the work for important intellectual content and final approval of the version to be published. AS: conception and design of the work; interpretation of data for the work; revising critically the work for important intellectual content and final approval of the version to be published. SX: conception and design of the work, acquisition, analysis and interpretation of data for the work; drafting and revising critically the work for important intellectual content and final approval of the version to be published.

## Conflict of Interest Statement

The authors declare that the research was conducted in the absence of any commercial or financial relationships that could be construed as a potential conflict of interest.

## Supplementary Material

FIGURE S1 Temporal order of pharmacological treatments. Treatment of cultures with **(A)** only CB_1_R and CB_2_R agonists, **(B)** CB_1_R or CB_2_R antagonists and CB_1_R or CB_2_R agonists or **(C)** CB_1_R and/or CB_2_R antagonists and CB_1_R and/or CB_2_R agonists.

FIGURE S2 Single cell calcium imaging (SCCI) timecourse and representative response patterns. Timecourse of SCCI experiments depicting the defined timepoints of stimulation with KCl and histamine (Hist) at 300 and 600 s, respectively. Representative [Ca^2+^]_i_ images of control, ACEA, HU-308, WIN55,212-2 and ACEA+HU-308 treated cultures at specific timepoints: baseline (120 s), KCl peak (300 s), KCl washout (480 s), Hist peak (600 s) and Hist washout (900 s); arrows indicate cells that display an increase in [Ca^2+^]_i_ in response to KCl but not to Hist stimulation representing neuronal-like cells.

## Abbreviations

2-AG: 2-arachidonoylglycerol
AEA: arachidonoylethanolamide
CBR: cannabinoid receptors
CB_1_R: cannabinoid receptors type 1
CB_2_R: cannabinoid receptors type 2
CNS: central nervous system
DG: dentate gyrus
EGF: epidermal growth factor
FGF-2: fibroblast growth factor-2
GPCRs: G protein-coupled receptors
NSPC: neural stem/progenitor cells
SVZ: subventricular zone
SGZ: subgranular zone
