# Supplementary figures and images for: Interaction between Cannabinoid Type 1 and Type 2 Receptors in the Modulation of Subventricular Zone and Dentate Gyrus Neurogenesis
Source: Front Pharmacol. 2017 Aug 10;8:516. doi: 10.3389/fphar.2017.00516 (PMC5554396; doi:10.3389/fphar.2017.00516)

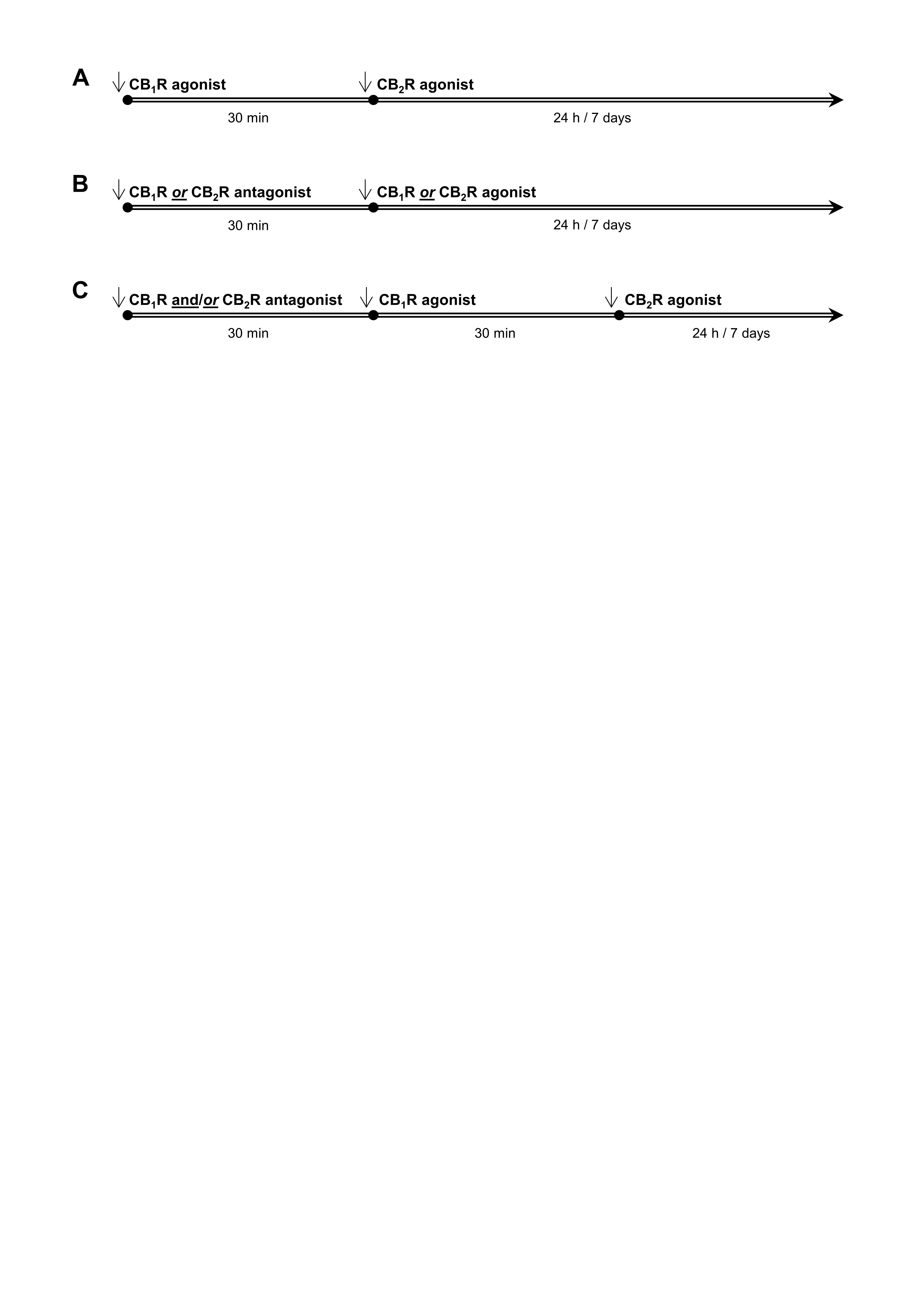

Supplement: FIGURE S1 — Temporal order of pharmacological treatments. Treatment of cultures with (A) only CB1R and CB2R agonists, (B) CB1R or CB2R antagonists and CB1R or CB2R agonists or (C) CB1R and/or CB2R antagonists and CB1R and/or CB2R agonists. [file Image_1.TIF]

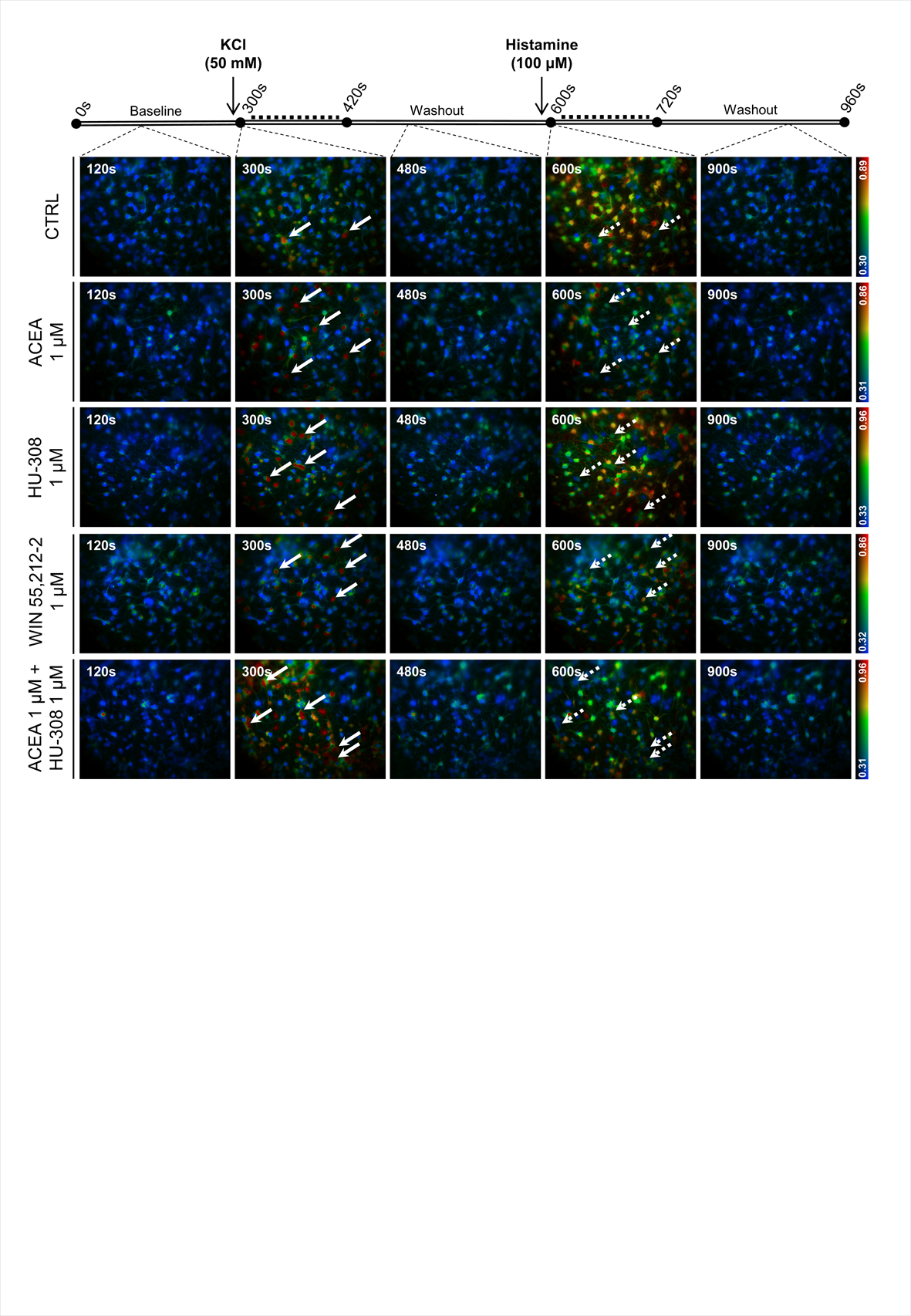

Supplement: FIGURE S2 — Single cell calcium imaging (SCCI) timecourse and representative response patterns. Timecourse of SCCI experiments depicting the defined timepoints of stimulation with KCl and histamine (Hist) at 300 and 600 s, respectively. Representative [Ca2+]i images of control, ACEA, HU-308, WIN55,212-2 and ACEA+HU-308 treated cultures at specific timepoints: baseline (120 s), KCl peak (300 s), KCl washout (480 s), Hist peak (600 s) and Hist washout (900 s); arrows indicate cells that display an increase in [Ca2+]i in response to KCl but not to Hist stimulation representing neuronal-like cells. [file Image_2.TIF]
